# Mitogenomic perspectives on the origin of Tibetan loaches and their adaptation to high altitude

**DOI:** 10.1038/srep29690

**Published:** 2016-07-15

**Authors:** Ying Wang, Yanjun Shen, Chenguang Feng, Kai Zhao, Zhaobin Song, Yanping Zhang, Liandong Yang, Shunping He

**Affiliations:** 1 The Key Laboratory of Aquatic Biodiversity and Conservation of Chinese Academy of Sciences, Institute of Hydrobiology, Chinese Academy of Sciences, Wuhan 430072, PR China; 2 University of the Chinese Academy of Sciences, Beijing 100049, PR China; 3 Key Laboratory of Adaptation and Evolution of Plateau Biota, Northwest Institute of Plateau Biology, Chinese Academy of Sciences, Xining 810001, China; 4 Sichuan Key Laboratory of Conservation Biology on Endangered Wildlife, College of Life Sciences, Sichuan University, Chengdu 610065, PR China; 5 Gansu Key Laboratory of Cold Water Fishes Germplasm Resources and Genetics Breeding, Gansu Fishers Research Institute, Lanzhou 730030, PR China

## Abstract

Tibetan loaches are the largest group of Tibetan fishes and are well adapted to the Tibetan Plateau. To investigate the origin of Tibetan loaches and their adaptations to the Tibetan Plateau, we determined 32 complete mitochondrial genomes that included 29 Tibetan loach species, two *Barbatula* species and *Schistura longus*. By combining these newly determined sequences with other previously published mitochondrial genomes, we assembled a large mitogenomic data set (11,433 bp) of 96 species in the superfamily Cobitoidea, to investigate the phylogenetic status of the genus *Triplophysa*. The resulting phylogeny strongly supported that the genus *Triplophysa* forms a monophyletic group within Nemacheilidae. Our molecular dating time suggests that the lineage leading to the Tibetan loaches and other loaches diverged approximately 23.5 Ma, which falls within the period of recent major uplifts of the Tibetan Plateau in the Early Miocene. Selection analyses revealed that the mitochondrial protein-coding genes of Tibetan loaches have larger ratios of nonsynonymous to synonymous substitutions than do those of non-Tibetan loaches, indicating that Tibetan loaches accumulated more nonsynonymous mutations than non-Tibetan loaches and exhibited rapid evolution. Two positively selected sites were identified in the *ATP8* and *ND1* genes.

Mitochondria are the energy metabolism centers of the cell and play critical roles in ATP synthesis and heat generation via cellular respiration. More than 95% of cellular energy is generated by mitochondria through oxidative phosphorylation (OXPHOS). Mitochondrial-encoded OXPHOS genes may therefore evolve under selection due to metabolic requirements and display evidence of adaptive evolution in mammals, birds and fishes^1^,^2^,^3^. Taxa subject to the cold and hypoxic conditions of high-altitude habitats have undergone alterations in mitochondrial function through changes in mitochondrial DNA^4^. Numerous studies have demonstrated the role of selection in mtDNA evolution and detected signals of positive selection in mitochondrial genes in endemic taxa of the Tibetan Plateau, including Tibetan humans^5^, the plateau pika^6^, the Tibetan horse^7^,^8^, the Tibetan antelope^9^, the Tibetan wild yak^10^, the Tibetan sheep^11^, Chinese snub-nosed monkeys^12^, galliform birds^13^ and schizothoracine fishes^14^. Mitochondria function in supplying cellular energy and are therefore extremely sensitive to energy-related selective pressure; furthermore, their small genome size, high substitution rate and easily accessible nature^15^ make them useful markers for phylogenetic reconstructions. Therefore, the mitogenome (mitochondrial genome) is widely used to not only explore phylogenetic relationships and estimate divergence times at different taxonomic levels^16^,^17^,^18^ but also detect signals of positive selection^2^,^3^,^10^.

The Superfamily Cobitoidea is comprised of seven monophyletic families Gyrinocheilidae, Catostomidae, Cobitidae, Botiidae, Vaillantellidae, Balitoridae and Nemacheilidae^19^. The Nemacheilidae is the largest group in the superfamily Cobitoidea, including numerous morphologically similar nemacheilid loaches^20^. Among the nemacheilid loaches, *Triplophysa* fishes have long been of great interest to researchers. The genus *Triplophysa* constitutes the largest of three major groups of Tibetan fishes and consists of 140 species reported in FishBase^21^, belonging to the family Nemacheilidae within the order Cypriniformes. *Triplophysa* species are widely distributed in the Tibetan Plateau and adjacent regions^22^. Studies of the morphological characteristics and geographical distribution of *Triplophysa* have suggested that the origin and evolution of this group is related to the uplift of the Tibetan Plateau^22^,^23^. The cold and hypoxic conditions of high-altitude habitats impose severe physiological challenges to organisms living in the Tibetan Plateau. As representative endemic species of the Tibetan Plateau, *Triplophysa* species are well adapted to the high-altitude environment. Nevertheless, except for some suggestions of mtDNA variation in *Triplophysa* associated with adaptation to high-altitude habitats^6^,^24^, an understanding of the genetic mechanisms that underlie the adaptations of this group to their high-altitude environment from a mitogenomic perspective is lacking. Moreover, the phylogenetic placement of *Triplophysa* and its divergence time from other nemacheilid loaches are not well understood.

In this study, we analyzed 32 complete, newly determined mitogenomes along with 64 published mitogenomes of the superfamily Cobitoidea, to 1) confirm the phylogenetic status of the genus *Triplophysa* within Nemacheilidae based on mitochondrial genomes and broad taxon sampling, 2) date the origin of the *Triplophysa* lineages, and 3) provide a comprehensive view of the adaptive evolution of the mitogenome in *Triplophysa* species during their independent acclimatization to high-altitude environments.

## Results

### Characteristics of the mitochondrial genome

The mitogenome sizes of the sequenced *Triplophysa* fishes ranged from 16,562 bp to 16,681 bp, and all of the mitogenomes exhibited similar sequence characteristics. Differences in mitogenome size resulted from variation in the length of the control regions. The gene arrangement, organization and content of the mitochondrial genome of *Triplophysa* fishes are similar to those of other teleosts^25^,^26^, which also contain 13 protein-coding genes, two ribosomal RNA genes (*rRNA*), 22 transfer RNA genes (*tRNA*), and a putative control region (CR). In the present study, the mitochondrial PCGs, tRNAs, rRNAs and CR were encoded on the heavy strand except for *ND6* and eight tRNA genes (*tRNA-Gln, tRNA-Ala, tRNA-Asn, tRNA-Cys, tRNA-Tyr, tRNA-Ser, tRNA-Glu*, and *tRNA-Pro*), which were encoded on the light strand. The concatenated data set consisted of 11,433 bp from 13 PCGs of 96 mitochondrial genomes. Of the identified sites, 6,081 (53.2%) sites were variable and 5,387 sites (47.1%) were parsimony informative. Additional details on the concatenated gene sequences and each of the 13 mitochondrial protein-coding genes (alignment length, variable sites, parsimony-informative sites, mean nucleotide composition, and transition/transversion ratios) are provided in Table 1. The examined mitochondrial genomes exhibited AT-bias (ranging from 52.4 to 58.5; average = 56.0).

### Phylogenetic status of the genus *Triplophysa*

Both the saturation plots and the Iss index values derived using DAMBE revealed no clear saturation for any codon position in the concatenated alignment (Figure S1). The combined data set of the 13 PCGs (total = 11,433 bp), including all codon positions, was used to conduct the phylogenetic analysis.

**Figure 1:**
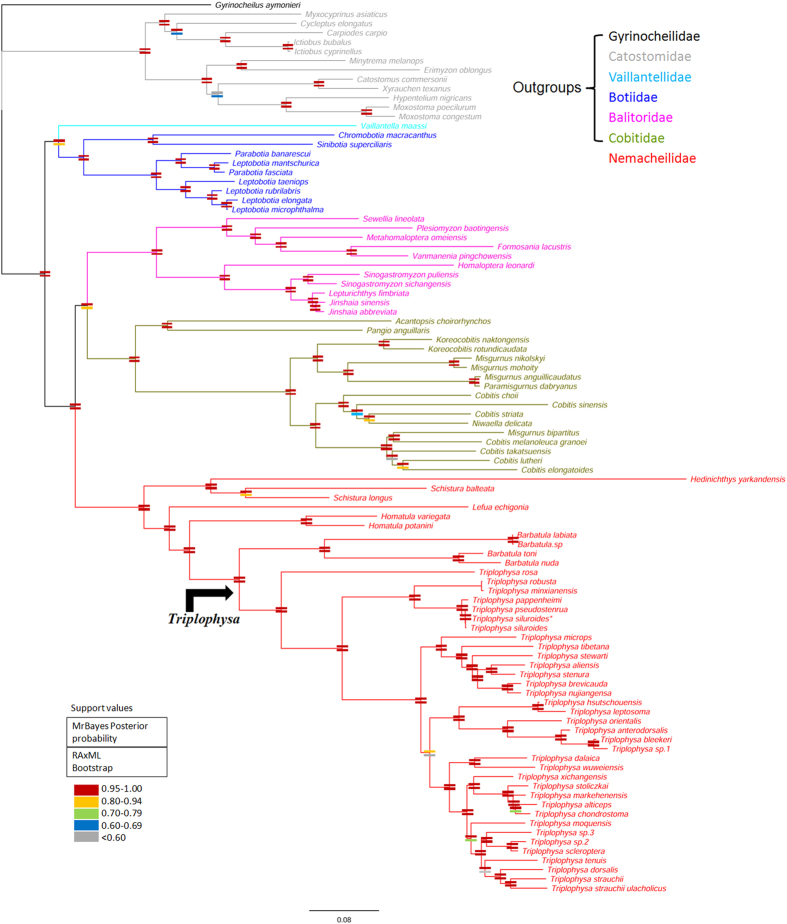
Phylogenetic trees of loaches inferred from the 96 mitogenomes of Cobitoidae, reconstructed using RAxML and Bayesian inference methods. At each node, the upper and lower rectangles with deferent colcor indicate the Bayesian posterior probability and the bootstrap value for the ML analyses, respectively. Branch lengths were estimated by using Bayesian inference method. (Scale bar represents 0.08 substitutions per site). Note: * represents the mitochondrial genome sequence has been sequenced in the previous study.

The ML and BI phylogenetic analyses of the concatenated data sets yielded consistent topological relationships for loaches with high bootstrap support values and Bayesian posterior probabilities (Fig. 1). Herein, ML bootstrap support values >70 and BI posterior probabilities >0.95 were defined as strongly supportive. The nodal support values obtained from two building methods are shown together on the BI topology (Fig. 1). The ML and BI trees strongly supported the monophyly of the loach clade. Within Cobitoidea, Gyrinocheilidae and Catostomidae rooted the phylogenetic tree. The remaining members of Cobitoidea represented a monophyletic group with strong nodal support and were resolved as ((Botiidae, Vaillantellidae), ((Cobitidae, Balitoridae), Nemacheilidae)). Vaillantellidae formed the sister group to the Botiidae clade with robust statistical support (posterior probability [PP] = 1.00 and bootstrap proportion [BP] = 81). Vaillantellidae and Botiidae formed the basal sister group to the other loaches and Cobitidae and Balitoridae formed a sister clade to Nemacheilidae. The above groups were used as outgroups while the family Nemacheilidae was used as the ingroups. Therefore, the family Nemacheilidae, especially the *Triplophysa* group is deserved our concern. A reasonably well-resolved phylogeny was yielded: the diverse nemacheilid loaches formed a monophyletic group with strong nodal support ([PP] = 1.00 and [BP] = 100), which comprised of 6 genera: *Acanthocobitis, Schistura, Lefua, Homatula, Barbatula* and *Triplophysa*. Each genera sampled formed its own monophyletic group, and most of the within-genera species relationships yielded high support values. The largest group, *Triplophysa*, was strongly supported as monophyletic ([PP] = 1.00 and [BP] = 100) with the exception of *Hedinichthys yarkandensis*, which is listed as a species of the subgenus *Hedinichthys*.

### Divergence time estimation for the *Triplophysa* lineage

**Figure 2:**
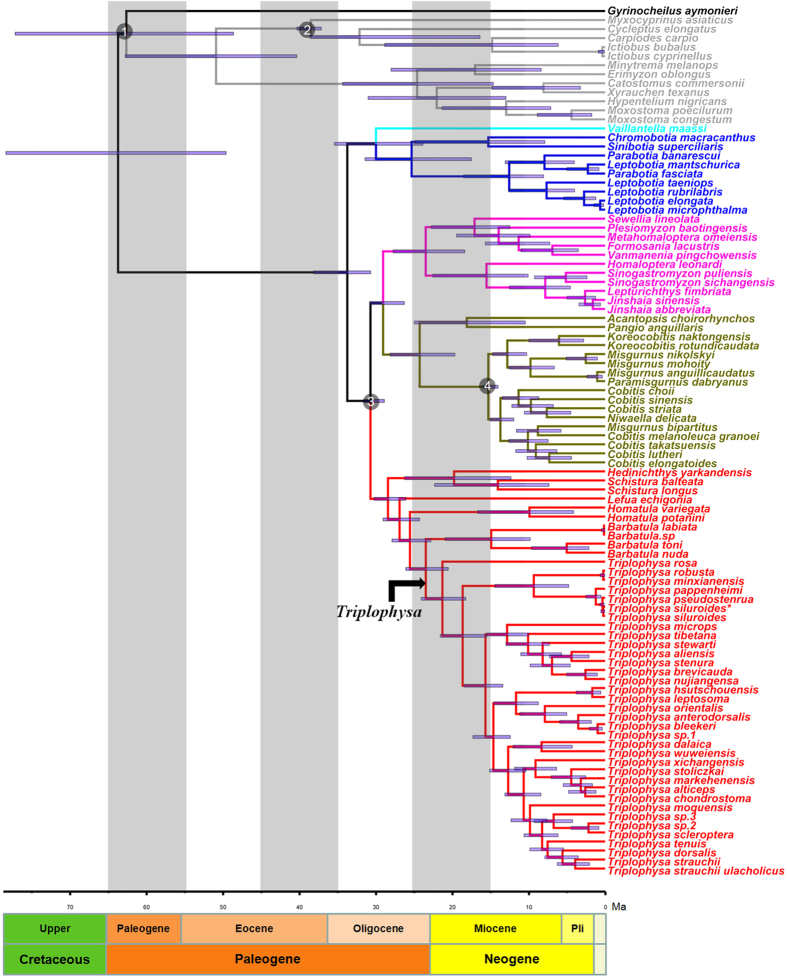
Divergence times among loaches derived from the Bayesian relaxed-molecular clock method. Numbers inside grey circles indicate the placement for the 4 calibrations used. Node bars indicate 95% credible intervals of the divergence time estimates.

We provided a timescale for loaches through MCMCTREE analysis. The estimated divergence times for loaches are shown in Fig. 2 with 95% credible intervals (CIs). The most recent common ancestor of loaches date to 33.8 Ma (95%CI: 30.7–38.2 Ma). The family Vaillantellidae diverged from the family Botiidae at 30 Ma (95%CI: 23.8–35.4 Ma), whereas the Balitoridae diverged from the Cobitidae at 29 Ma (95%CI: 26.3–30.8 Ma). The most recent common ancestor of Nemacheilidae date to 28 Ma (95%CI: 26.1–30.2 Ma). The adaptive radiation of *Triplophysa* fishes is a major event in the evolution of nemacheilid loaches, with *Triplophysa* being the largest group within the Nemacheilidae. This group diverged from the other loaches within the Nemacheilidae at 23.5 Ma (95%CI: 20.5–26.1 Ma), and the most recent common ancestor of *Triplophysa* group arose 21.3 Ma (95%CI: 18.2–24.1 Ma).

### Selection analysis

**Figure 3:**
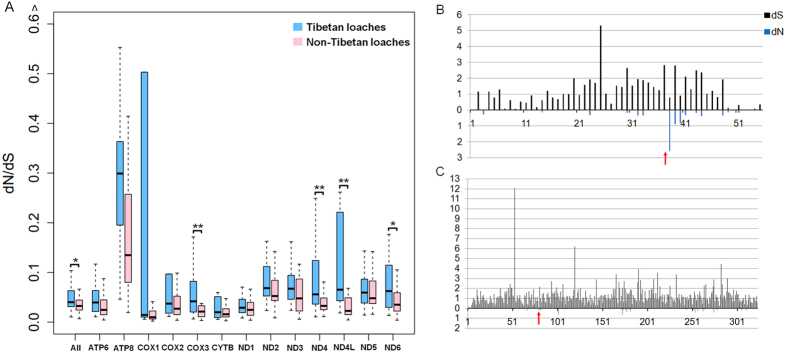
Selection pressure analysis and the positive genes identified for *Triplophysa* fishes. (**A**) Comparisons of average *dN/dS* ratios for 13 individual protein-coding genes between *Triplophysa* fishes and Non-*Triplophysa* fishes. Note: *0.01 < P < 0.05, **0.001 < P < 0.01. (**B**) *dN* and *dS* estimated for the positive gene *ATP8* using the FEL analysis method. The red arrow indicates the position of positive selected site. (**C**) *dN* and *dS* estimated for the positive gene *ND1* using the FEL analysis method. The red arrow indicates the position of positive selected site.

Selection analysis yielded a separate dN/dS ratio for each terminal branch of the phylogenetic tree (Table S1). The dN/dS ratio of Tibetan loaches was significantly larger than that of non-Tibetan loaches for the 13 concatenated mitochondrial protein-coding genes (Wilcoxon rank sum test, P = 0.03016). Moreover, each of the 13 mitochondrial protein-coding genes had a larger dN/dS ratio in the Tibetan loaches than in the non-Tibetan loaches (Fig. 3). In particular, the *dN/dS* ratios of *COX1, ND4, ND4L*, and *ND6* in the Tibetan loaches were significantly larger than those in the non-Tibetan loaches (Wilcoxon rank sum test, P = 0.004286, 0.00044, 0.007778, and 0.02895, respectively) among all of the mitochondrial protein-coding genes. These results implied that Tibetan loaches experienced weaker purifying selection at mitochondrial protein-coding genes than did non-Tibetan loaches and that the former accumulated more nonsynonymous mutations.

The FEL analysis identified two positively selected sites in the *ATP8* (corresponding to site 38, *dN/dS* = 3.228154003, P = 0.027923387) and *ND1* (corresponding to site 80, *dN/dS* = 6.933321774, P = 0.049226799) genes (Fig. 3, Table S2).

## Discussion

The loaches are generally recognized as comprising five families, i.e., Botiidae, Vaillantellidae, Cobitidae, Balitoridae, and Nemacheilidae, according to morphological characters^27^,^28^ and molecular data^19^,^29^. As a diverse group, the phylogenetic relationships of loaches have been studied extensively in the order Cypriniformes^18^,^19^,^30^,^31^. Nevertheless, previous studies did not recover a comprehensively consistent phylogenetic tree. Slechtova *et al*.^19^ confirmed the family status of the Vaillantellidae, but some discrepancies remained regarding its placement: Vaillantellidae, Botiidae or both were found to occupy the basal position among the loaches^18^,^19^,^25^,^31^. The present analysis strongly supports the clustering of Vaillantellidae and Botiidae as a basal clade within the loach families. The remaining loaches (Cobitidae, Balitoridae, and Nemacheilidae) have been clustered into a single clade^18^,^30^, which is supported by our analysis. However, conflicting results have been obtained regarding the sister-group relationships among these three families. A sister relationship between Balitoridae and Nemacheilidae is supported by some previous studies^18^,^19^,^30^, whereas a sister relationship between Cobitidae and Nemacheilidae was proposed by Tang *et al*.^20^. However, all of these studies involved a limited number of taxa or genes. For example, only one species of Balitoridae, *Homaloptera leonardi*, was included in the study by Mayden *et al*. 2009, and a single *RAG1* gene was used for the phylogenetic reconstruction of Cobitoidea by Slechtova *et al*.^19^. Therefore, compared with the taxon data of previous studies, our study incorporated the largest data set of loach mitochondrial genomes. In the present study, the sister relationship between Cobitidae and Balitoridae is resolved, and Nemacheilidae forms a monophyletic group. Our analysis clustered *Hedinichthys* with *Schistura* within Nemacheilidae instead of with *Triplophysa*, which indicates that *Hedinichthys yarkandensis* is not a genuine Tibetan loach. He *et al*.^32^, found that *Hedinichthys* clustered with the genus *Lefua* based on analysis of the *CYTB* gene. We suggest that the placement of *Hedinichthys* should be redefined. The genus *Triplophysa* should exclude the subgenus *Hedinichthys*, recovering the genus *Triplophysa* as a monophyletic group.

A previous study found a correlation between the geological and biotic evolution of the Tibetan Plateau in a paleobiogeographical analysis of freshwater fishes^33^. Therefore, it is important for researchers to consider the divergence time of *Triplophysa* within the context of the geological history of the Tibetan Plateau. Previously, He *et al*.^32^ concluded that *Triplophysa* diverged from the other loaches of the family Nemacheilidae 13.5–10.3 Ma ago based on strict molecular clock estimation. Wang *et al*.^24^, proposed that *Triplophysa rosa* diverged from other *Triplophysa* species approximately 48.3 Ma (34.7–59.5 Ma) ago based on analysis of combined *CYTB* and D-loop sequences. Our age estimation for *Triplophysa* is incongruent with the above molecular clock estimations. Our molecular dating results indicate that the most recent common ancestor of *Triplophysa* fishes diverged from other nemacheilid loaches approximately 23.5 Ma (95% CI: 20.5–26.1 Ma). With respect to the origin of schizothoracine fishes, Ruber *et al*.^34^ revealed the common ancestor of the schizothoracine fishes was in the Oligocene-Miocene boundary (around 23 Ma) or older based on the relaxed molecular clock analysis of cyprinids from cytochrome b. Therefore, the molecular dating time of the Tibetan loach lineage is consistent with that of the schizothoracine fishes. Tibetan loaches are mainly distributed in the high-altitude lakes of the Himalayas. Considering this distribution and our divergence time estimates, we hypothesize that the formation of this distribution pattern is likely associated with the concomitant ecological changes that occurred during the Tibetan Plateau uplift process. It is reported that southern Tibet and the Himalayas began to uplift due to rapid crustal thickening in the Early Miocene approximately 21–17 Ma ago^35^,^36^,^37^. Our estimate of the origin of *Triplophysa* is compatible with the timing of the geological events that occurred during the rapid uplift in Tibet. Therefore, we assume that the uplift of the Tibetan Plateau played an important role in the speciation of *Triplophysa* fishes in the Early Miocene. At the same time, the divergence date estimates of *Triplophysa* fishes might reflect the occurrence of geological events associated with the uplift of plateau. The rapid and persistent rise of the Tibetan Plateau began approximately 8 Ma^38^; its ultimate height did not lead to the extinction of *Triplophysa* but rather its adaptation to the extreme environment.

Previously, Sun *et al*.^3^ proposed that mitochondrial genes have undergone adaptive evolution in teleosts because of their different metabolic requirements. They divided the mitochondrial data set of 401 fishes into “migratory” and “nonmigratory” groups and tested functional constraints act on mitochondria. In comparision, the size of data sets (96 complete mitochondrial genomes) in this study is smaller than that of the previous study. Nevertheless, our results also detected the significant difference between the *dN/dS* ratios of Tibetan loaches and the non-Tibetan loaches. Compared with the non-Tibetan loaches (ω = 0.04834), the Tibetan loaches (ω = 0.10665) had a significantly larger mean ω (*dN/dS*) ratio for the 13 concatenated mitochondrial protein-coding genes, indicating that the high-altitude groups have accumulated more nonsynonymous mutations. These nonsynonymous mutations have resulted in slightly beneficial amino acid changes that allowed adaption to the high-altitude environments. Individually, the 13 mitochondrial protein-coding genes were also shown to have larger *dN/dS* ratios in the Tibetan loaches than in the non-Tibetan loaches, which provide consistent evidence for accelerated evolution at the mitogenome level in Tibetan loaches compared with non-Tibetan loaches. A previous study of galliform birds also found a larger mean ω (*dN/dS*) ratio for 13 concatenated mitochondrial protein-coding genes in the branches of high-altitude birds^13^. Previously, we found evidence of genome-wide, rapid evolution of Tibetan loaches relative to fishes living at low altitudes from our analysis of Tibetan loach transcriptome data^39^. Tibetan loaches adapted well to the severe conditions of the Tibetan Plateau by means of accelerated evolutionary rates. Martin and Palumbi^40^ proposed that the larger *dN/dS* in mitochondrial genes could be related to some physiological variables, such as metabolic rate. Considering the distribution of *Triplophysa* and our divergence time estimate, these results suggest that the evolutionary rate of *Triplophysa* fishes might be influenced by the geological events of the Tibetan Plateau uplift in the Early Miocene.

Among the 13 mitochondrial protein-coding genes involved in oxidative phosphorylation (OXPHOS), *ND1* and *ATP8* have undergone positive selection. *ND1* is one of seven subunits in NADH dehydrogenase; it is the first and largest enzyme complex and acts as a proton pump^41^,^42^. *ATP8* is one of two subunits in ATP synthase; it is the last enzyme complex and uses a concentration gradient of protons to produce ATP^43^,^44^. Numerous studies have indicated that the adaptive evolution of the NADH dehydrogenase complex and ATP synthase has been vital in the evolution of energy generation by oxidative phosphorylation^8^,^13^,^14^,^41^,^45^. We suggest that both the *ND1* and *ATP8* genes are responsible for high-altitude adaptation in *Triplophysa* fishes. Similarly, with respect to the high altitude adaptation in schizothoracine fishes, Li *et al*.^14^ found the positively selected sites in *ND1* gene. On the contrary, they also detected the positively selected sites in *ATP6, CYTB, ND2, ND4* and *ND5* genes. These results suggested that Tibetan loaches and schizothoracine fishes probably employ different genic toolkit to adapt to the extreme environment of the Tibetan Plateau.

## Materials and Methods

All the methods were carried out in accordance with approved guidelines. All experimental protocols involving animals in this study were approved by the Ethics Committee of the Institute of Hydrobiology, Chinese Academy of Sciences.

### Specimen collection and DNA extraction

Specimen information for 29 *Triplophysa* species, two *Barbatula* species, and *Schistura longus* is provided in Table 2. The species identification was following the previous book^22^. All of the samples were stored in 95% ethanol until DNA extraction. Total genomic DNA was extracted from muscle or fin tissue using a standard phenol/chloroform extraction method^46^.

### Mitochondrial genome sequencing, assembly and annotation

The 17 PCR amplification primer sets used for the mitogenomes are described in Table S3. The primers were designed from the conserved regions identified based on the alignment of 15 complete mitogenomes available for Nemacheilidae (www.ncbi.nlm.nih.gov). PCR was carried out in 30 μl reaction volumes containing 19.7 μl sterilized distilled water, 4 μl of 10 × PCR buffer II (Takara, China), 2.0 μl dNTP (2.5 mM), 1.5 μl of each primer (10 μM), 0.3 μl of Taq (5U/μl Takara, China), and 1.0 μl of template DNA (appropriate 30 ng). The cycling protocol was as follows: initial denaturation at 95 °C for 4 min followed by 30 cycles of 94 °C for 30 s, 50–55 °C for 40 s, and 72 °C for 2 min, with a final extension at 72 °C for 10 min. PCR products were electrophoresed on a 1.0% agarose gel, purified with a DNA Agarose Gel Extraction Kit (Omega, USA) and sequenced using an ABI3730xl sequencer (Applied Biosystems, Foster City, CA, USA).

The sequences were edited and assembled using the SeqMan program from the Lasergene package (DNASTAR Inc., Madison, WI, USA). The protein-coding genes, *rRNA* genes, *tRNA* genes and non-coding regions of mitogenomes were annotated using MitoAnnotator^47^. Newly determined mitogenome sequences were deposited in GenBank (Table S4).

### Phylogenetic reconstruction

Including the 32 complete mitogenomes that were newly determined in this study, a total of 96 mitochondrial genomes were used in the phylogenetic analysis, including the outgroup Catostomidae and Gyrinocheilidae. The taxonomic information, accession numbers and mitogenome sizes are provided in Table S4. The nucleotide sequences of the 13 protein-coding genes (PCGs) were first extracted using purpose-built perl scripts^48^ based on annotations and then separately aligned according to their corresponding amino acid translations using the software TranslatorX^49^. The gaps were not removed as they are known to contain valuable information for phylogenetic analyses^50^. The concatenated nucleotide sequence alignment from 13 PCGs (total = 11,433 bp) without stop codons was generated with our in-house scripts to conduct the phylogenetic analysis. Tests of the base substitution saturation were performed prior to the phylogenetic reconstruction. The extent of substitution saturation was estimated separately for entire codons and for the first, second, and third codon positions of the concatenated alignment using DAMBE^51^. The pairwise nucleotide differences (transitions and transversions) were plotted against the GTR genetic distance.

The best-fit global model GTR+I+G was selected for the alignment of the 13 concatenated PCGs based on the Bayesian information criterion (BIC) using jModelTest2.1.3^52^. To improve the reliability of the phylogenetic analysis, the best-fit partitioning scheme across each gene and codon position was determined for each data set under the Bayesian information criterion using PartitionFinder software^53^.

Both partitioned maximum likelihood (ML) and Bayesian Inference (BI) approaches with the selected partition scheme (Table S5) were employed to reconstruct the phylogenetic relationships among the loach families. We implemented the Bayesian phylogenies in MrBayes v3.2.3^54^,^55^ with the “unlink” and “prest ratepr = variable” model parameters. Two independent runs were performed with four independent Markov Chain Monte Carlo (MCMC) chains (three hot and one cold) for 50,000,000 generations initiated from a random tree, sampling one tree every 1000 generations. Convergence of the BI analyses was first assessed by the average standard deviation of split frequencies less than 0.01 and the potential scale reduction factors (PSRF) close to 1.0 for all parameters. We also used Tracer v1.6 software^56^ to investigate the convergence of the BI analyses. The first 12,500 trees were discarded as conservative burn-in, and the remaining samples were used to generate a majority-rule consensus tree. The support values of the BI tree were estimated by Bayesian posterior probability (BPP). The ML phylogenetic analysis was implemented in RAxML v8.1.17^57^ with the GTRGAMMAI model. 1,000 rapid bootstrapping replications were used to evaluate the bootstrap support values of the ML phylogenetic tree and search for the best-scoring ML tree.

### Divergence time estimation

MCMCTREE in PAML v4.8 was implemented to estimate divergence times with an approximate likelihood calculation^58^. The ML phylogenetic tree topology from the 13 concatenated PCGs was used for divergence time estimation, and the ML branch lengths were estimated using the BASEML program in PAML under the GTR substitution model with the gamma prior set at 0.5. Two priors, the overall substitution rate (rgene gamma) and rate-drift parameter (sigma2 gamma), were set at G (1, 4.5) and G (1, 1.52), respectively, using the strict molecular-clock assumption with a root age of 152 Ma, which is suggested by previous studies^59^ (http://www.timetree.org/search/pairwise/). The independent rates model (clock = 2 in MCMCTREE)^60^ was used to specify the prior of rates among the internal nodes, which followed a log-normal distribution. The three parameters (birth rate λ, death rate μ and sampling fraction ρ) in the birth-death process with species sampling were specified as 1, 1, and 0, respectively. A loose maximum bound for the root was set at >0.658 <1.800 (i.e., between 65.8 Ma and 180 Ma).

The following four fossil calibrations were incorporated in this study: 1) The minimum age of Catostomidae is 60 Ma based on a catostomid fossil from the Paleocene^61^. 2) The oldest fossil of *Plesiomyxocyprinus arratiae* similar to *Myxocyprinus asiaticus* was constrained to from the middle Eocene or earlier, approximately 38–40 Ma^62^. 3) The estimated divergence time between Cobitinae and Nemacheilinae is approximately 30 Ma^63^. 4) The fossil calibrations of the genus *Cobitis* were recorded as 13.8–15.9 Ma^64^ (http://www.wahre-staerke.com/). The first 200,000 cycles in MCMCTREE were discarded as burn-in, and every 50 cycles were sampled to obtain a total of 20,000 samples. To ensure that convergence was reached, two replicate MCMC runs were initiated with two different random seeds. The distributions of the parameter values from the MCMC samples were assessed using Tracer v1.6 (ESS > 200)^56^.

### Analysis of selective pressure

The codeml program in the PAML package^58^ with the free ratio model (model = 1) was used to estimate the ratio of nonsynonymous (*dN*) to synonymous (*dS*) substitutions rates (ω = *dN/dS*), which allowed for a separate *dN/dS* ratio for each branch on a tree. From the optimized ML tree topology derived from the 13 concatenated protein-coding genes, the *dN/dS* ratios of the 13 concatenated mitochondrial protein-coding genes and the 13 individual genes were computed separately for the terminal branches to evaluate the selective pressure operating on the mtDNA genomes. The *dN/dS* ratios for each terminal branch were divided into two groups: Tibetan loaches and non-Tibetan loaches. We tested the statistical significance of the differences in the *dN/dS* ratios of each gene between the Tibetan and non-Tibetan loaches using the Wilcoxon rank sum test implemented in R v2.10.0^65^.

To identify the genes that have undergone positive selection for high-altitude adaptation, a branch model and a branch site model were employed to detect significant changes in selective pressure. However, the results of likelihood ratio tests (LRTs) for each mitochondrial gene were not significant. Therefore, the fixed-effects likelihood (FEL) approach implemented in HyPhy software^66^ was used to detect site-specific selection pressure; this approach is more powerful than the codeml program for detecting individual sites subject to episodic diversifying selection^67^. The FEL approach was run using the best-fitting nucleotide substitution model for each gene that was identified by jModelTest2.1.3^52^ on the ML phylogenetic tree. The subtree consisting of the 35 Tibetan loaches was specified as the foreground branch and tested while the rest of the branches shared an arbitrary *dN/dS* ratio. Two models were nested in this method: *H*_*0*_, *dN* = *dS* (the neutral model) and *H*_*A*_, where *dN* and *dS* are estimated independently (the selection model). A nominal significance level of 0.1 for the likelihood ratio test was chosen based on the desired power of this analysis. When the LRT is significant, if *dN* > *dS*, the site is declared to be under positive selection, otherwise the site is under negative selection.

## Additional Information

**How to cite this article**: Wang, Y. *et al*. Mitogenomic perspectives on the origin of Tibetan loaches and their adaptation to high altitude. *Sci. Rep.* **6**, 29690; doi: 10.1038/srep29690 (2016).

## Supplementary Material

Supplementary Information

## Figures and Tables

**Table 1:** Characterization of datasets used in this study.

Genes	Alignment length	Parsimony informative sites	Variable sites	Nucleotide composition (%)				Ti/Tv
				T	C	A	G	
Combined data	11433	5387	6081	29.3	27.0	26.8	16.9	1.98
*ATP6*	705	318	405	31.2	27.3	27.0	14.6	1.77
*ATP8*	165	87	117	26.0	28.5	32.5	13.0	2.11
*COX1*	1539	590	649	30.5	25.5	25.3	18.7	2.08
*COX2*	690	281	316	28.2	25.9	28.9	17.0	2.42
*COX3*	783	317	350	28.3	27.8	26.3	17.6	2.08
*CYTB*	1134	513	557	29.6	27.6	26.9	15.9	2.09
*ND1*	972	451	500	28.6	28.4	26.5	16.5	1.89
*ND2*	1044	604	647	25.8	30.1	29.1	15.0	1.67
*ND3*	348	181	190	31.2	27.4	24.8	16.5	2.64
*ND4*	1380	680	787	28.7	27.5	28.0	15.8	2.04
*ND4L*	294	135	150	28.9	30.2	23.5	17.3	2.04
*ND5*	1860	968	1113	28.7	27.6	28.8	14.9	1.99
*ND6*	519	262	300	38.4	14.6	15.9	31.1	2.23

Note: Ti = transition; Tv = transversion.

**Table 2:** Specimen information for sequenced species.

Species	Species-voucher	Location
*Triplophysa siluroides*	IHB201308861	Gansu
*Triplophysa stenura*	IHB201305756	Gongshan province, Yunnan
*Triplophysa nujiangensa*	IHB201315814	Fugong county, Yunnan
*Triplophysa sp.3*	IHB201308867	Ertix River, Xinjiang
*Triplophysa sp.1*	IHB0908447	Sichuan
*Triplophysa scleroptera*	IHB201306600	Baijia wholesale market of aquatic products, Chengdu
*Triplophysa dalaica*	IHB201306599	Baijia wholesale market of aquatic products, Chengdu
*Triplophysa wuweiensis*	IHB201307124	Jinchuanxia reservoir in Yongchang county, Gansu
*Triplophysa tenuis*	IHB201307126	Dang river in Subei county, Gansu
*Triplophysa minxianensis*	IHB0917490	Niutou river in Qingshui county, Gansu
*Triplophysa hsutschouensis*	IHB201307128	Sunai county, Gansu
*Triplophysa stewarti*	IHB201306547	Langcuo, Tibet
*Triplophysa microps*	IHB201306545	Changchenmo river, Tibet
*Triplophysa strauchii ulacholicus*	IHB201305179	Mulei river, Xinjiang
*Triplophysa aliensis*	NWIPB1106031	Mafamu lake, Xinjiang
*Triplophysa tibetana*	NWIPB1106069	Mafamu lake, Xinjiang
*Triplophysa alticeps*	NWIPB1206002	Qihai lake
*Triplophysa leptosoma*	NWIPB1109002	Ganzi river, Qinghai
*Triplophysa pappenheimi*	NWIPB1250383	Dari county, Qinghai
*Triplophysa pseudostenrua*	NWIPB20070704	Yalong river, Sichuan
*Hedinichthys yarkandensis*	NWIPB1007001	Cheerchen river, Qinghai
*Triplophysa moquensis*	SCU20130901	Ruoergai river in Xiaman village, Sichuan
*Triplophysa chondrostoma*	NWIPB1006052	Tiangeli river, Qinghai
*Triplophysa dorsalis*	NWIPB1305230	Tekesi river in Tekesi county, Qinghai
*Triplophysa brevicauda*	SCU20090621	Muli county, Sichuan
*Triplophysa markehenensis*	SCU1010706	Sichuan Taimen Protective Center in Qinghai Provincial Fishery Environmental Monitoring Center
*Triplophysa orientalis*	SCU20070912	Zequ tributary of Dadu River in Rangtang county, Sichuan
*Triplophysa sp.2*	IHB201306570	Anning tributary of Chishui river, Sichuan
*Triplophysa xichangensis*	IHB201306572	Anning tributary of Chishui river, Sichuan
*Barbatula labiata*	IHB201306569	Xinyuan county, Xinjiang
*Barbatula sp.*	IHB201306543	Xinyuan county, Xinjiang
*Schistura longus*	IHB00915689	Pi river in Fugong county, Yunnan

Note: IHB, Institute of Hydrobiology, Chinese Academy of Sciences; SCU, Sichuan University; NWIPB, Northwest Institute of Plateau Biology, Chinese Academy of Sciences.
