# Supplementary material for: Mitogenomic perspectives on the origin of Tibetan loaches and their adaptation to high altitude
Source: Sci Rep. 2016 Jul 15;6:29690. doi: 10.1038/srep29690 (PMC4945904; doi:10.1038/srep29690)

Supplementary information:

Mitogenomic perspectives on the origin of Tibetan loaches and their adaptation to high altitude

Ying Wang[1,2], Yanjun Shen[1,2], Chenguang Feng[2,3], Kai Zhao[3], Zhaobin Song[4], Yanping Zhang[5], Liandong Yang[1], Shunping He[1*]

[1]The Key Laboratory of Aquatic Biodiversity and Conservation of Chinese Academy of Sciences, Institute of Hydrobiology, Chinese Academy of Sciences, Wuhan 430072, PR China

[2]University of the Chinese Academy of Sciences, Beijing 100049, PR China

[3]Key Laboratory of Adaptation and Evolution of Plateau Biota, Northwest Institute of Plateau Biology, Chinese Academy of Sciences, Xining 810001, China

[4]Sichuan Key Laboratory of Conservation Biology on Endangered Wildlife, College of Life Sciences, Sichuan University, Chengdu 610065, PR China

[5]Gansu Key Laboratory of Cold Water Fishes Germplasm Resources and Genetics Breeding, Gansu Fishers Research Institute, Lanzhou 730030, PR China

*Correspondence: S.P. He. The Key Laboratory of Aquatic Biodiversity and Conservation of Chinese Academy of Sciences, Institute of Hydrobiology, Chinese Academy of Sciences, Wuhan 430072, PR China

E-mail addresses: clad@ihb.ac.cn

Table S1: The dN/dS ratios for concatenated 13 mitochondrial protein-coding genes and individual genes.

Table S2: Selection analyses for ATP8 and ND1 genes.

Table S3. Primers designed for the mitogenome amplifications.

Table S4. List of the species used in this study, with Genbank accession numbers and mitogenome sizes.

Table S5. Partition scheme used in this study.

Figure S1. Saturation tests for any codon position in the concatenated alignment.

TableS1: The dN/dS ratios for concatenated 13 mitochondrial protein-coding genes and individual genes.

Group	Species	Concatenated Data	ATP6	ATP8	COX1	COX2	COX3	CYTB	ND1	ND2	ND3	ND4	ND4L	ND5	ND6
	Myxocyprinus asiaticus	0.0199	0.0044	0.0207	0.0001	0.0001	0.0001	0.0082	0.009	0.0427	0.009	0.0252	0.0001	0.0341	0.0123
	Barbatula labiata	30.6439	0.0001	2.9109	0.0001	2.2172	0.0001	2.7309	0.0001	0.0001	3.5379	0.0001	30.799	905.2989	610.5869
	Formosania lacustris	0.037	0.0296	0.1335	0.0154	0.0218	0.0227	0.013	0.0389	0.0868	0.0299	0.0531	0.0485	0.052	0.0148
	Cobitis striata	0.0242	0.0117	0.1864	0.0001	0.0001	0.0001	0.0108	0.0348	0.0738	0.0131	0.02	0.0001	0.0415	0.0546
	Lefua echigonia	0.0323	0.0131	0.0795	0.0032	0.0174	0.0236	0.026	0.024	0.0559	0.0347	0.0283	0.0495	0.044	0.031
	Carpiodes carpio	0.0173	0.0001	119.1648	0.0088	0.0099	0.0132	0.0001	0.0204	0.0085	0.0001	0.0185	0.0001	0.0297	0.0223
	Cobitis sinensis	0.0806	0.2481	2.379	0.0205	0.027	0.0001	0.0125	0.0132	0.0874	0.0347	0.0529	0.1374	0.1186	0.0229
	Minytrema melanops	0.0214	0.0001	0.0001	0.0001	0.0107	0.008	0.0032	0.0316	0.0611	0.0163	0.0256	0.0001	0.0421	0.0039
	Cycleptus elongatus	0.0273	0.0001	0.6435	0.0027	0.0101	0.0001	0.013	0.0205	0.0682	0.049	0.044	0.0619	0.0401	0.011
	Catostomus commersonii	0.0252	0.0001	0.0001	0.0101	0.0001	0.0214	0.016	0.0066	0.0634	0.0001	0.0486	967.5703	0.0391	0.0085
	Acantopsis choirorhynchos	0.0275	0.0057	0.123	0.0056	0.0313	0.0116	0.009	0.0129	0.0493	0.043	0.0192	0.0069	0.0408	0.0163
	Barbatula toni	0.0231	0.0567	0.0001	0.0001	1.3701	0.0001	0.0174	0.0079	0.0154	0.0001	0.0446	0.0001	0.0418	0.0001
	Chromobotia macracanthus	0.0288	0.0238	0.1543	0.003	0.0187	0.0254	0.0158	0.0137	0.0843	0.0057	0.0189	0.0001	0.0469	0.0293
	Gyrinocheilus aymonieri	0.0239	0.017	0.0507	0.0062	0.0001	0.011	0.0109	0.0038	0.0454	0.0102	0.0148	0.0001	0.0511	0.0565
	Homaloptera leonardi	0.0664	0.0303	0.1265	0.0075	0.0114	0.021	0.0268	0.0846	0.1172	0.0867	0.0664	0.0681	0.1158	0.0164
	Moxostoma poecilurum	0.0397	0.0001	0.164	0.0001	0.0988	0.0366	0.0412	0.063	0.0485	0.0001	0.0508	0.0001	0.0355	0.0001
	Pangio anguillaris	0.0177	0.0042	0.023	0.0028	0.0001	0.0059	0.0055	0.0071	0.0333	0.058	0.0197	0.0165	0.023	0.0407
	Hypentelium nigricans	0.0195	0.0072	0.0675	0.0001	0.0001	0.0092	0.0169	0.0207	0.0452	0.0224	0.0414	0.0001	0.0181	0.0223
	Leptobotia mantschurica	0.014	0.0001	0.0001	0.0001	0.0001	0.0001	0.0001	0.024	0.0641	0.0001	0.0631	0.0001	0.0001	0.0001
	Misgurnus nikolskyi	0.0198	0.0001	0.0001	0.0001	0.0001	0.0001	0.0398	0.0001	0.0231	0.0001	0.0142	0.0001	0.0481	0.0351
	Schistura balteata	0.0385	0.0411	0.1348	0.0128	0.0038	0.03	0.0255	0.026	0.0453	0.2049	0.0328	0.0077	0.0455	0.0753
	Vaillantella maassi	0.0548	0.0459	0.2049	0.0099	0.0214	0.0358	0.0364	0.0502	0.0515	0.0478	0.0261	0.0197	0.0679	0.0812
	Xyrauchen texanus	0.0639	0.2447	0.0532	0.0078	0.0001	0.0001	0.0082	0.0247	0.0525	0.0735	0.0446	0.0001	0.1359	0.0402
	Cobitis choii	0.0277	0.0067	0.1945	0.0001	0.0127	0.0089	0.0025	0.0412	0.05	0.1166	0.0316	0.0127	0.0471	0.0647
	Misgurnus anguillicaudatus	0.086	0.7076	0.0001	0.0576	0.2799	0.0001	0.0001	0.0452	0.0001	0.0001	0.0477	0.0001	0.3379	0.0001
	Sinogastromyzon puliensis	0.0333	0.0211	0.2866	0.0264	0.0001	0.0094	0.0139	0.0171	0.0428	0.0669	0.0293	0.0001	0.0914	0.029
	Erimyzon oblongus	0.0256	0.0235	0.0398	0.0001	0.0001	0.0001	0.0116	0.0158	0.031	0.0225	0.0274	0.0001	0.0528	0.0199
	Ictiobus bubalus	0.0371	0.0001	0.0001	0.0001	1.4928	0.0001	0.1685	0.0001	0.0001	2.2298	0.0001	0.0001	0.0001	0.0001
	Moxostoma congestum	0.0382	0.0001	0.1706	0.0001	0.0167	0.0001	0.0148	0.0397	0.1422	0.0001	0.0612	0.0001	0.083	0.0001
	Cobitis takatsuensis	0.0447	0.0335	0.4143	0.0214	0.037	0.0001	0.0542	0.0348	0.1273	0.0128	0.0325	0.0218	0.0547	0.0861
non-Tibetan loaches	*Niwaella delicata*	0.0298	0.061	0.6844	0.0098	0.0776	0.0155	0.0042	0.0123	0.0445	0.0702	0.0213	0.0136	0.0167	0.0661
	Sewellia lineolata	0.0265	0.0144	0.1071	0.0087	0.0196	0.0033	0.004	0.0292	0.0493	0.0104	0.0375	0.0226	0.0504	0.0458
	Koreocobitis naktongensis	0.0533	0.0208	0.0001	0.0884	0.0001	0.0237	0.0314	0.041	0.1092	0.0947	0.058	0.0001	0.0326	0.1986
	Koreocobitis rotundicaudata	0.0244	0.0437	0.0001	0.0062	1.1486	0.02	0.0001	0.0093	0.0526	0.0316	0.026	0.0001	0.0323	0.0429
	Leptobotia elongata	0.0652	0.0001	0.0001	0.0144	0.0428	0.0001	2.5052	999	0.0001	0.0001	0.0001	6.7648	0.0861	0.0001
	Homatula variegata	0.0347	0.0154	0.1298	0.0001	0.0165	0.0342	0.027	0.0055	0.0978	0.06	0.0361	0.0505	0.0425	0.0222
	Sinibotia superciliaris	0.039	0.026	0.0393	0.0065	0.0244	0.0306	0.0269	0.0332	0.049	0.0407	0.0276	0.0271	0.0706	0.0517
	Misgurnus mohoity	0.0411	0.0001	0.2281	0.0224	0.0001	0.0001	0.0475	0.0315	0.0946	0.2003	0.081	0.0001	0.0385	0.0001
	Cobitis lutheri	0.0459	0.0587	0.0001	0.0406	0.134	0.0333	0.0204	0.0152	0.0539	0.0218	0.0266	0.0001	0.0929	0.0564
	Leptobotia rubrilabris	0.0212	0.0001	0.0001	2.202	2.1518	0.0001	0.0104	0.0001	1.7999	0.9844	0.0178	6.0636	0.1421	0.0001
	Misgurnus bipartitus	0.0345	0.0182	0.3078	0.0099	0.0137	0.0176	0.0055	0.0199	0.0873	0.1166	0.0435	0.0001	0.0523	0.0332
	Barbatula nuda	0.0335	0.0296	0.0001	0.0076	0.0001	0.0153	0.0092	0.0327	0.0379	0.094	0.0449	0.0001	0.1133	0.0001
	Plesiomyzon baotingensis	0.0278	0.0253	0.0001	0.0021	0.0098	0.0371	0.0168	0.0159	0.0501	0.0307	0.0273	0.0184	0.0312	0.0154
	Cobitis melanoleuca granoei	0.0359	0.0323	0.0001	0.0001	0.0518	0.0206	0.0133	0.0632	0.0743	0.016	0.0475	0.0248	0.0533	0.036
	Paramisgurnus dabryanus	0.0282	0.0001	0.0001	0.0001	2.1394	0.0001	0.0001	0.0001	0.0001	0.0001	0.0001	0.0001	0.2871	0.0001
	Cobitis elongatoides	0.1088	0.1095	0.37	0.0051	0.0443	0.034	0.0158	0.1039	0.1685	0.0904	0.1476	0.0001	0.1842	0.1858
	Leptobotia microphthalma	0.5773	449.7387	0.3286	2.0258	1.9919	0.0001	1.2021	0.0001	1.4181	1.1404	0.0001	6.4849	0.0001	0.0001
	Sinogastromyzon sichangensis	0.0326	0.0247	0.0001	0.0126	0.0001	0.033	0.0559	0.0001	0.0413	0.0001	0.0311	0.0001	0.0328	0.035
	Jinshaia sinensis	0.1263	0.1154	0.5847	0.2868	0.0001	0.0001	0.0001	0.4526	0.0001	0.0001	0.1466	0.0001	0.0652	0.0001
	Jinshaia abbreviata	0.0509	0.0001	0.0001	0.0411	0.0001	0.0001	0.0775	0.052	0.0409	0.0785	0.0001	0.0001	0.1122	0.1053
	Lepturichthys fimbriata	0.0099	0.0001	0.0001	0.0001	0.0001	0.0001	0.0001	0.0001	0.0001	0.1058	0.0369	0.0001	0.018	0.0001
	Metahomaloptera omeiensis	0.044	0.0076	0.5933	0.0411	0.0429	0.0001	0.0134	0.0001	0.0505	3.1962	0.0723	0.0001	0.0422	0.0225
	Homatula potanini	0.0348	0.0207	0.0807	0.0106	0.0001	0.0312	0.0142	0.0315	0.0544	0.0501	0.012	0.0001	0.0969	0.0591
	Parabotia banarescui	0.0068	0.0001	0.0001	0.0001	0.0001	0.0001	0.0216	0.0001	0.0001	0.0001	0.0111	0.0001	0.0159	0.0718
	Parabotia fasciata	0.0306	0.0001	0.1859	0.0001	0.0001	0.0001	0.04	0.0297	0.0264	0.0001	0.0393	0.0001	0.0516	0.0001
	Leptobotia taeniops	0.0244	0.0001	0.0818	0.0051	0.0391	0.0001	0.0083	0.0664	0.0418	0.0634	0.0202	0.0001	0.0362	0.0685
	Vanmanenia pingchowensis	0.0523	0.0875	0.1142	0.0504	0.0349	0.0376	0.018	0.0233	0.084	3.156	0.0519	0.0001	0.0537	0.0297
	Ictiobus cyprinellus	0.175	0.0001	0.0001	0.0001	0.0001	0.1904	2.0731	0.0001	0.0001	3.216	0.2478	0.0001	999	999
	Hedinichthys yarkandensis	0.0177	0.0129	0.0192	0.0028	0.0071	0.0044	0.003	0.0051	0.0221	0.014	0.0143	0.0043	0.0277	0.0077
	Schistura longus	0.0289	0.0188	0.0001	0.0001	0.0526	0.0105	0.0198	0.0066	0.0773	0.0239	0.03	0.0259	0.0826	0.0001
	Barbatula sp.	0.0238	0.0001	7.5913	1.3691	0.0001	0.0001	2.6357	0.2602	0.0001	3.4801	0.0001	37.3862	0.0001	999
	Triplophysa anterodorsalis	0.1036	0.0001	4.8908	0.0001	0.0656	0.1715	0.0286	64.007	0.1148	5.4879	44.7928	21.2445	924.7574	0.0241
	Triplophysa bleekeri	0.1652	0.0001	7.2945	0.5031	0.8546	0.1681	0.0413	0.0001	0.066	0.0001	999	0.0001	999	0.0001
	Triplophysa robusta	1.4695	0.0001	0.0001	1.4904	1.924	0.0001	2.1779	0.3525	2.3966	4.6404	0.0001	1.8499	999	0.6641
	Triplophysa rosa	0.0611	0.0415	0.1952	0.0152	0.0447	0.0874	0.0596	0.0157	0.0641	0.0331	0.0907	0.0431	0.0644	0.0922
	Triplophysa siluroides	0.3427	68.1295	0.0001	0.0001	0.0001	0.3765	0.5783	0.0001	0.4498	6.0802	0.2472	999	0.4409	1.6552
	Triplophysa stoliczkai	0.019	0.0001	0.0001	0.0001	0.0001	0.0001	0.0001	0.0217	0.0233	0.0001	0.0285	0.2616	0.0427	0.0001
	Triplophysa strauchii	0.0364	0.059	0.0001	0.01	0.0001	0.0409	0.0084	0.0223	0.109	0.0432	0.0323	0.1234	0.0419	0.045
	Triplophysa aliensis	0.0343	0.0392	0.0001	0.0001	0.0001	0.052	0.0001	0.0273	0.0759	0.0673	0.0362	0.0184	0.0237	0.0297
	Triplophysa alticeps	0.0168	0.0001	0.0001	0.0001	0.0001	0.0418	0.0001	0.0327	0.0001	0.0001	0.0264	0.0001	0.0142	0.1146
	Triplophysa sp.1	0.0383	0.0001	0.2078	0.0001	0.0001	0.0001	0.0001	0.0483	0.0784	0.0001	0.058	0.0001	0.0822	0.0955
	Triplophysa sp.2	0.0692	0.0001	0.3634	0.0001	0.0001	0.0769	0.0001	0.0001	0.1441	0.1063	0.1241	0.0001	0.0945	0.1767
	Triplophysa brevicauda	0.0444	0.0683	0.0001	0.0001	0.0001	0.0001	0.0001	0.0001	0.0646	0.0001	0.0676	0.0001	0.0817	0.1422
	Triplophysa chondrostoma	0.0445	0.0001	0.0001	0.0001	0.0967	0.0001	0.0164	0.0001	0.0626	0.0001	0.1142	0.0001	0.0836	0.0001
	Triplophysa dalaica	0.0227	0.0001	0.0001	0.0001	0.0001	0.0001	0.0056	0.0193	0.0565	0.1098	0.0304	0.0001	0.0566	0.0001
	Triplophysa dorsalis	0.03	0.0136	0.0001	0.0001	0.018	0.0185	0.0124	0.0429	0.0592	0.0939	0.0515	0.0001	0.0328	0.0001
	Triplophysa hsutschouensis	0.3224	0.0925	0.0001	0.0001	2.2964	0.062	0.0001	0.0001	5.3044	6.8492	0.4014	0.0001	0.0001	0.0001
	Triplophysa leptosoma	0.0654	0.0182	0.0725	0.0001	2.3377	0.0063	1.7673	0.0001	0.0001	6.8637	0.0001	0.0001	999	0.0001
Tibetan loaches	*Triplophysa markehenensis*	0.0257	0.0001	0.0001	0.0001	0.0001	0.0408	0.0295	0.0306	0.0356	0.0001	0.0302	0.0001	0.0455	0.0001
	Triplophysa microps	0.0326	0.0218	0.2991	0.0111	0.0001	0.0085	0.0092	0.0306	0.0759	0.0357	0.0416	0.0223	0.0374	0.0256
	Triplophysa minxianensis	0.1623	0.0001	0.0001	0.0001	1.7278	0.0001	0.2306	0.0001	0.496	4.5293	0.6297	0.0001	0.1695	0.0001
	Triplophysa moquensis	0.0198	0.0001	0.0001	0.0083	0.0001	0.0001	0.0223	0.0138	0.0431	0.0237	0.015	0.0533	0.0309	0.0001
	Triplophysa nujiangensa	0.0401	0.0788	0.0001	0.0001	0.0001	0.0672	0.0001	0.0704	0.0486	5.7938	0.5009	17.4879	0.0526	0.0624
	Triplophysa orientalis	0.0364	0.0388	232.4631	0.0001	0.0001	0.0001	0.0513	0.0083	0.0354	0.1618	0.0479	0.2211	0.0891	0.0001
	Triplophysa pappenheimi	0.0333	999	0.0001	0.0001	0.0159	0.0001	0.0001	0.0001	0.0001	0.0001	0.0001	18.9524	0.1433	0.0001
	Triplophysa pseudostenrua	0.1221	0.0001	0.0001	0.0001	0.0001	0.1874	0.0001	0.0001	0.0001	0.0001	0.1665	28.4157	0.4423	0.0001
	Triplophysa scleroptera	0.0392	0.1168	0.0001	0.0001	0.0001	0.0001	0.116	0.0001	0.1197	0.0001	0.2354	0.0001	0.0001	0.0001
	Triplophysa siluroides	0.0557	0.0001	0.0001	1.961	2.032	0.0001	0.0001	0.0001	0.0001	6.1216	0.249	22.1633	0.0001	89.4288
	Triplophysa stenura	0.0104	0.0001	0.0001	0.0001	0.0001	0.0001	0.0001	0.013	0.0335	5.7656	999	21.5519	0.0181	0.0001
	Triplophysa stewarti	0.0417	0.0395	0.0459	0.0001	0.0147	0.0227	0.0068	0.0245	0.0708	0.0906	0.0928	0.0607	0.0496	0.055
	Triplophysa sp.3	0.0483	0.0001	0.0001	0.0058	0.0001	0.016	0.0072	0.047	0.1077	0.0912	0.1179	0.0699	0.0891	0.0388
	Triplophysa strauchii ulacholicus	0.0245	0.0001	0.5528	0.0001	0.0001	0.0001	0.0001	0.0392	0.0443	0.0001	0.0533	0.0001	0.0387	0.0222
	Triplophysa tenuis	0.0434	0.0496	0.3669	0.0147	0.0258	0.0131	0.0199	0.0459	0.1467	0.0626	0.0105	0.0001	0.0622	0.0133
	Triplophysa tibetana	0.0499	0.0108	0.0001	0.0142	0.0115	0.022	0.0167	0.0627	0.1627	0.0942	0.0511	0.0001	0.0648	0.065
	Triplophysa wuweiensis	0.0372	0.021	0.3626	0.0001	0.0362	0.0001	0.0154	0.0191	0.0804	0.0485	0.0421	0.0001	0.0723	0.0001
	Triplophysa xichangensis	0.0245	0.0001	0.0001	0.0001	0.0372	0.0001	0.0084	0.0097	0.063	0.0521	0.0559	0.0001	0.0381	0.0001

TableS2: Selection analyses for *ATP8* and *ND1* genes.

ATP8 _FEL.out_positive_selection

Codon posistion	dN	dS	dN/dS	dS=dN	LRT	p-value	Full Log(L)	dN_other
1	0	0	0	0	0	1	0	0
2	0	1.172818	0	0.745291	11.83383	0.000582	-46.997456	0
3	0.270359	0	inf	0.103208	2.119686	0.145416	-8.6212364	0
4	0	1.165961	0	0.866912	8.515609	0.003521	-65.659276	0
5	0	0.804866	0	0.526886	7.009987	0.008106	-34.809306	0.023612
6	0	1.310271	0	0.85258	13.15901	0.000286	-57.058557	0
7	0	0.097502	0	0.068562	1.201166	0.273089	-42.948183	0.196998
8	0	0.634451	0	0.432399	7.076306	0.007811	-42.711714	0
9	0	0.090437	0	0.064181	0.652195	0.419329	-12.107372	0.067449
10	0	0.545178	0	0.330089	6.653466	0.009896	-37.872626	0.036142
11	0	0.482331	0	0.264705	5.007884	0.025232	-56.962658	0.268551
12	0	0.932432	0	0.520935	9.775401	0.001769	-39.501077	0.019764
13	0	0.19414	0	0.160217	1.699998	0.192288	-17.986907	0
14	0.141068	0.634374	0.222373	0.434477	2.473301	0.115794	-65.067065	0.194825
15	0	1.226355	0	0.645856	13.09983	0.000295	-49.143003	0.016982
16	0	0.799473	0	0.462254	7.590757	0.005867	-32.595669	0.02011
17	0	0.699063	0	0.328031	3.821162	0.050609	-26.553438	0.097999
18	0	1.021474	0	0.794705	7.342003	0.006736	-107.22793	0.382851
19	0	0.992831	0	0.540626	10.12515	0.001463	-62.477435	0.156286
20	0	1.993797	0	0.988488	19.19311	1.18E-05	-52.536625	0.016741
21	0	0.963502	0	0.677616	10.87262	0.000976	-48.834216	0
22	0	1.589475	0	1.033483	13.81429	0.000202	-97.794932	0.334388
23	0.307986	1.932329	0.159386	1.28074	7.212308	0.007241	-103.8704	0.272399
24	0	1.710412	0	0.898314	15.52805	8.13E-05	-58.877202	0.067683
25	0	5.328806	0	2.211437	32.37654	1.27E-08	-95.613444	0.019102
26	0	1.033401	0	0.689645	9.700714	0.001842	-59.028146	0.12543
27	0	0.40847	0	0.218624	2.272734	0.131667	-17.891865	0.038042
28	0	1.554596	0	0.904103	15.46709	8.40E-05	-74.397344	0.095081
29	0	1.446626	0	0.81054	13.77416	0.000206	-69.318144	0.086789
30	0.162545	2.653701	0.061252	1.444695	13.67199	0.000218	-91.174417	0.334294
31	0	1.540528	0	0.886044	13.23157	0.000275	-50.113438	0.025415
32	0.326766	1.948096	0.167736	1.212908	6.80413	0.009095	-119.79767	0.598395
33	0.326126	1.889939	0.172559	1.245451	5.763018	0.016367	-134.96747	0.491624
34	0	1.733078	0	0.987395	15.60546	7.80E-05	-62.228085	0.023279
35	0	1.460642	0	0.899793	11.78179	0.000598	-56.409338	0.024563
36	0	1.252469	0	0.848568	11.46195	0.00071	-71.50164	0.116118
37	0	2.839404	0	1.379496	21.55789	3.43E-06	-72.561007	0.047786
38	2.563622	0.794145	3.228154	1.259522	4.832791	0.027923	-131.04132	0.414191
39	0.877767	2.807582	0.312641	2.034435	4.8649	0.027408	-114.19927	0.177609
40	0.80403	0.908145	0.885354	0.882559	0.040416	0.840669	-126.20328	0.388902
41	0.314986	2.106443	0.149535	1.28874	7.904889	0.00493	-85.805823	0.258094
42	0	1.322011	0	0.895726	13.4059	0.000251	-95.542199	0.353748
43	0.160548	2.508498	0.064002	1.002372	9.591069	0.001955	-71.40834	0.145675
44	0.372511	2.366886	0.157384	1.182257	6.33817	0.011817	-69.515515	0.31469
45	0	1.034155	0	0.633261	9.602583	0.001943	-37.759095	0
46	0	1.206403	0	0.569282	5.88855	0.01524	-29.164955	0.035144
47	0	0.817715	0	0.583714	8.140037	0.00433	-71.48673	0.106032
48	0.337408	1.935814	0.174298	0.842635	3.967829	0.046378	-52.064445	0.056812
49	0.140517	0.153184	0.917308	0.149385	0.00521	0.942457	-44.560374	0.169188
50	0	0	0	0	0	1	0	0
51	0.154229	0.325802	0.473382	0.26401	0.477064	0.489755	-47.450992	0.135512

52	0	0	0	0	0	1	0	0
53	0	0	0	0	0	1	0	0
54	0	0.090337	0	0.078425	0.679924	0.409613	-18.172279	0
55	0	0.35694	0	0.209822	1.940295	0.163637	-10.7948	0

Note: The positively selected residue is coloured red.

ND1 _FEL.out_positive_selection

The codon posis	dN	dS	dN/dS	dS=dN	LRT	p-value	Full Log(L)	dN_other
1	0	0	#NAME?	0	0	1	-9.4126515	0.01484
2	0	0.158544	0	0.125867	2.642992	0.104007	-57.595705	0.10287
3	0	0	#NAME?	0	0	1	-22.427157	0.046096
4	0	0.060084	0	0.037156	1.911498	0.166797	-35.234083	0.058721
5	0	0.316238	0	0.254164	5.230879	0.022189	-62.688073	0.034251
6	0.095269	0.571882	0.166588	0.429371	4.73412	0.02957	-94.787296	0.099961
7	0	1.023821	0	0.474321	25.08891	5.47E-07	-96.710401	0.080972
8	0	0.863831	0	0.396043	17.48946	2.89E-05	-75.31534	0.113073
9	0	0.807091	0	0.617545	12.74163	0.000358	-95.509086	0
10	0.118783	0.370029	0.321011	0.267056	2.462871	0.116566	-64.969474	0.061362
11	0	1.865368	0	0.620569	35.26099	2.88E-09	-52.176128	0
12	0	1.206388	0	0.663556	27.39914	1.66E-07	-87.889535	0
13	0	0.742987	0	0.487064	16.27878	5.47E-05	-81.207101	0.022455
14	0	0.448331	0	0.231051	12.04548	0.000519	-65.347795	0.060129
15	0	1.719629	0	0.663046	27.42502	1.63E-07	-55.68935	0
16	0.053655	0.824372	0.065086	0.416252	13.42377	0.000248	-49.59048	0
17	0.109421	1.179742	0.09275	0.645221	16.63443	4.53E-05	-123.2588	0.199787
18	0	1.315145	0	0.63774	27.83013	1.32E-07	-75.186525	0
19	0	0.96124	0	0.50628	21.83242	2.98E-06	-81.516869	0.033511
20	0	0.719632	0	0.563712	11.43639	0.00072	-97.482413	0
21	0	0.790556	0	0.580166	12.67765	0.00037	-96.96307	0.029828
22	0	0.840485	0	0.416842	21.35446	3.82E-06	-69.303957	0
23	0	0.468982	0	0.289549	12.15066	0.000491	-55.313601	0.016726
24	0	0.400564	0	0.220867	11.23003	0.000805	-47.05518	0
25	0	0.618799	0	0.305293	14.26782	0.000159	-34.832051	0
26	0	1.254892	0	0.718148	24.54337	7.27E-07	-92.939579	0.025124
27	0	0.886556	0	0.380451	20.92892	4.77E-06	-63.329486	0
28	0	0.558407	0	0.375423	12.59263	0.000387	-73.460486	0
29	0	0.527913	0	0.330938	11.39007	0.000738	-95.396828	0.166727
30	0	1.419704	0	0.568761	28.11643	1.14E-07	-59.509759	0.016256
31	0	0.250457	0	0.147228	6.055907	0.01386	-31.576284	0
32	0	0.492953	0	0.22763	9.050789	0.002626	-29.302398	0
33	0	1.035104	0	0.540071	23.36958	1.34E-06	-72.759204	0
34	0	1.153186	0	0.804008	17.24824	3.28E-05	-105.08136	0.030009
35	0	0.952833	0	0.531652	25.11357	5.41E-07	-83.674016	0.019405
36	0	1.212532	0	0.574514	22.46467	2.14E-06	-60.729081	0
37	0	1.979398	0	0.537844	32.44863	1.22E-08	-56.659809	0.014827
38	0	1.226631	0	0.435476	13.89684	0.000193	-40.369297	0
39	0	0.999419	0	0.700606	15.24694	9.43E-05	-106.96404	0.029484
40	0	0.928431	0	0.521834	19.96073	7.90E-06	-77.221338	0
41	0	0.5334	0	0.288263	10.74672	0.001045	-41.521032	0
42	0	1.657622	0	0.983537	27.39553	1.66E-07	-102.35663	0
43	0	1.427794	0	0.561997	26.41627	2.75E-07	-71.636133	0
44	0	1.685731	0	0.531499	31.08938	2.46E-08	-59.742033	0
45	0	0.41265	0	0.211292	11.34328	0.000757	-61.720851	0.050063

46	0	2.023867	0	0.842307	37.00835	1.18E-09	-86.878035	0
47	0	1.123008	0	0.603907	17.73007	2.55E-05	-69.10651	0
48	0	2.444995	0	0.858441	40.3767	2.09E-10	-90.437624	0
49	0	2.062793	0	0.81961	33.17547	8.42E-09	-58.461707	0
50	0	2.031619	0	0.961944	29.54476	5.46E-08	-85.835785	0
51	0	1.285295	0	0.780129	25.59952	4.20E-07	-85.660221	0
52	0	1.15849	0	0.707281	23.30991	1.38E-06	-83.791048	0
53	0	12.04933	0	0.767507	34.22324	4.91E-09	-44.997398	0
54	0	0.801006	0	0.385794	17.36763	3.08E-05	-58.097726	0
55	0	0.577421	0	0.276729	15.11286	0.000101	-37.297224	0
56	0	1.292365	0	0.666258	32.44866	1.22E-08	-86.257942	0
57	0	0.911321	0	0.425992	21.71426	3.16E-06	-45.069949	0
58	0	0.967493	0	0.678223	17.43716	2.97E-05	-87.962695	0
59	0	1.317494	0	0.606893	26.83808	2.21E-07	-90.557992	0.033948
60	0	0.791046	0	0.316419	13.05314	0.000303	-33.248272	0
61	0	0.589081	0	0.446866	9.435212	0.002129	-73.118886	0
62	0	0.406067	0	0.2236	10.08638	0.001494	-34.451669	0
63	0	0.258177	0	0.137173	7.33124	0.006777	-37.298175	0.014338
64	0	1.327928	0	0.429965	18.83845	1.42E-05	-40.490097	0
65	0	1.158389	0	0.49918	23.93967	9.94E-07	-52.595645	0
66	0	1.552219	0	0.626607	29.1992	6.53E-08	-77.285977	0
67	0.244403	0.395777	0.617526	0.324103	0.573546	0.448853	-73.697928	0.081098
68	0	0.338513	0	0.207799	8.396293	0.00376	-41.779586	0
69	0	1.712088	0	0.729146	32.70456	1.07E-08	-80.693852	0
70	0	1.117231	0	0.559606	25.55989	4.29E-07	-71.646046	0
71	0	0.828455	0	0.379937	20.41311	6.24E-06	-76.839234	0.029198
72	0.059511	1.383081	0.043028	0.600426	19.5875	9.61E-06	-90.752416	0.033831
73	0	0.293753	0	0.176456	7.851219	0.005079	-37.458492	0
74	0	1.892775	0	0.742362	34.03424	5.42E-09	-88.730428	0
75	0.058545	0.450395	0.129986	0.264369	5.800221	0.016024	-76.634909	0.104308
76	0	1.120114	0	0.773743	17.65948	2.64E-05	-98.92649	0
77	0	0.758476	0	0.378823	17.41425	3.01E-05	-43.820264	0
78	0	1.145	0	0.79734	17.56271	2.78E-05	-103.58666	0.036787
79	0.053116	0.709092	0.074907	0.374716	11.58182	0.000666	-91.70195	0.128939
80	0.227838	0.032861	6.933322	0.104897	3.867602	0.049227	-62.702462	0.140164
81	0	2.147231	0	0.915163	40.02423	2.51E-10	-89.679654	0
82	0.2149	1.001023	0.214681	0.514823	7.052782	0.007914	-95.478707	0.076779
83	0	0.616136	0	0.40144	11.66486	0.000637	-77.916818	0.021987
84	0	1.429372	0	0.531636	29.73568	4.95E-08	-77.016561	0
85	0	1.521056	0	1.009834	22.46913	2.14E-06	-129.41353	0.105414
86	0	1.048006	0	0.417298	23.33777	1.36E-06	-83.198663	0.028981
87	0	0.639851	0	0.510612	10.59017	0.001137	-89.857558	0
88	0	0.541747	0	0.305927	15.22292	9.55E-05	-57.944875	0.014744
89	0.054467	1.39609	0.039014	0.536576	19.451	1.03E-05	-91.78226	0.063818
90	0	1.560192	0	0.680276	33.47806	7.21E-09	-100.03653	0.061241
91	0	1.069923	0	0.740248	16.07853	6.08E-05	-105.88917	0.028852
92	0	1.14027	0	0.414519	14.17454	0.000167	-38.918275	0
93	0.051158	0.485423	0.105389	0.271446	7.614105	0.005791	-58.85511	0.014799
94	0	2.726182	0	1.049171	46.77106	7.98E-12	-89.281859	0
95	0	1.593352	0	0.534058	29.98038	4.36E-08	-71.609834	0.044734
96	0	1.449806	0	0.678772	29.69941	5.05E-08	-81.869551	0
97	0	1.472185	0	0.51889	28.29433	1.04E-07	-56.747757	0
98	0	1.724103	0	0.750392	33.64293	6.62E-09	-82.895237	0
99	0	1.089238	0	0.612001	16.72752	4.32E-05	-92.95061	0.160955

100	0	2.065389	0	0.785763	36.51558	1.51E-09	-82.159692	0
101	0	1.569026	0	0.829633	33.64731	6.61E-09	-101.529	0.033996
102	0.656059	1.159208	0.565954	0.881269	1.629852	0.201724	-124.06811	0.090692
103	0	0.851301	0	0.345668	18.68241	1.54E-05	-57.428058	0.030871
104	0	1.666848	0	1.058695	24.21578	8.61E-07	-121.47341	0.025271
105	0	0.698921	0	0.317239	17.23525	3.30E-05	-40.472245	0
106	0	1.119979	0	0.826074	18.00184	2.21E-05	-114.37626	0
107	0	2.385416	0	1.10579	38.66423	5.03E-10	-97.028224	0
108	0.102384	1.127132	0.090836	0.516463	14.78596	0.00012	-91.014311	0.093939
109	0	0.906618	0	0.665801	15.04976	0.000105	-96.987315	0
110	0	0.355321	0	0.19795	8.905671	0.002843	-32.125584	0
111	0.120463	1.24712	0.096593	0.678245	15.26559	9.34E-05	-138.97397	0.257441
112	0	1.187818	0	0.883199	17.86619	2.37E-05	-119.72436	0
113	0	0.716206	0	0.356652	18.47502	1.72E-05	-64.304015	0
114	0	1.242583	0	0.81688	17.67641	2.62E-05	-117.07773	0.054196
115	0	1.019562	0	0.458051	21.77143	3.07E-06	-71.933115	0
116	0	0.848492	0	0.350785	19.78534	8.66E-06	-46.668757	0
117	0	0.925953	0	0.664459	14.01623	0.000181	-85.065918	0
118	0	1.235909	0	0.557918	29.03805	7.10E-08	-71.1029	0
119	0	1.829082	0	0.804476	34.74862	3.75E-09	-90.924274	0
120	0	6.201117	0	0.900664	41.69939	1.06E-10	-71.212905	0.019535
121	0.057528	0.965103	0.059608	0.504617	15.3742	8.82E-05	-91.294657	0.033924
122	0	1.044262	0	0.452078	24.75415	6.51E-07	-66.048367	0.014798
123	0	1.568673	0	1.003926	23.21384	1.45E-06	-120.73664	0.025694
124	0	1.89222	0	0.82776	31.73684	1.77E-08	-101.58038	0
125	0	0.872438	0	0.42294	18.95048	1.34E-05	-75.512743	0.016809
126	0	2.019694	0	0.967813	28.65909	8.63E-08	-112.08409	0.019862
127	0	0.834236	0	0.346949	11.45805	0.000712	-46.408421	0.024781
128	0	0.595902	0	0.272734	14.80168	0.000119	-52.093433	0
129	0	1.031378	0	0.485034	22.76951	1.83E-06	-63.904835	0
130	0	1.024217	0	0.427106	23.44166	1.29E-06	-48.372943	0
131	0	0.253014	0	0.144627	6.29213	0.012128	-32.331886	0
132	0	1.780235	0	0.54854	23.4845	1.26E-06	-44.680175	0
133	0	1.09569	0	0.536302	20.89994	4.84E-06	-53.51762	0
134	0	0.55294	0	0.266245	14.1604	0.000168	-54.869012	0
135	0	0.839719	0	0.621128	12.91193	0.000326	-96.638122	0.028666
136	0	0.659979	0	0.320168	17.25484	3.27E-05	-45.376022	0
137	0	1.358897	0	0.675494	32.37087	1.27E-08	-96.595393	0.019263
138	0.051169	0.338857	0.151005	0.215485	4.911149	0.026684	-50.513247	0
139	0	0.978883	0	0.64379	20.44026	6.15E-06	-92.306586	0
140	0	0.800785	0	0.527859	18.8158	1.44E-05	-72.430619	0
141	0	0.506442	0	0.282793	14.18554	0.000166	-48.195256	0
142	0	1.232735	0	0.618421	26.61191	2.49E-07	-73.349907	0
143	0	0.300886	0	0.173654	8.731869	0.003127	-35.632817	0
144	0	2.867868	0	0.921665	29.3377	6.08E-08	-53.738022	0
145	0	0.306942	0	0.159967	8.486357	0.003578	-34.243363	0
146	0	0.912756	0	0.409766	22.34153	2.28E-06	-54.853907	0
147	0	1.097696	0	0.502664	23.05902	1.57E-06	-71.421356	0
148	0	0.806444	0	0.429267	16.38837	5.16E-05	-62.741892	0.03931
149	0	1.26744	0	0.55914	26.63431	2.46E-07	-53.104196	0
150	0	1.269185	0	0.56368	25.67209	4.05E-07	-68.299554	0
151	0	0.538618	0	0.241156	13.46122	0.000244	-34.331694	0
152	0	1.186219	0	0.708526	23.7676	1.09E-06	-95.321526	0
153	0	1.322644	0	0.803424	22.63632	1.96E-06	-84.754738	0

154	0	1.352201	0	0.854872	19.97852	7.83E-06	-116.56254	0.029363
155	0	1.001491	0	0.462484	24.78673	6.40E-07	-48.142184	0
156	0	0.8791	0	0.67704	15.09094	0.000102	-111.80461	0
157	0	1.398475	0	0.894108	21.08673	4.39E-06	-118.17279	0.027078
158	0	0.993673	0	0.472977	22.24704	2.40E-06	-77.956805	0.016789
159	0.154824	0.915141	0.16918	0.501493	9.76364	0.00178	-113.20871	0.167776
160	0	0.884046	0	0.404255	21.89273	2.88E-06	-66.037998	0.029405
161	0.050277	1.391952	0.03612	0.531779	22.24445	2.40E-06	-67.396197	0.029057
162	0	1.070436	0	0.490024	22.92872	1.68E-06	-45.900211	0
163	0.05889	1.89687	0.031046	0.851078	29.38206	5.94E-08	-115.44688	0.088142
164	0	1.157991	0	0.760485	20.11421	7.30E-06	-96.927941	0
165	0	1.951434	0	0.86866	39.84078	2.76E-10	-99.901778	0
166	0	3.42052	0	0.80476	34.96671	3.35E-09	-63.124835	0
167	0	0.95128	0	0.404157	22.81731	1.78E-06	-67.181571	0
168	0	0.529012	0	0.395912	9.60937	0.001936	-90.027537	0.029051
169	0	2.60767	0	0.660724	22.7084	1.89E-06	-51.970956	0.026092
170	0	1.980932	0	0.530204	31.07632	2.48E-08	-92.557212	0.114675
171	0	0.676529	0	0.360663	16.22543	5.62E-05	-49.372365	0.016652
172	0.103896	1.234734	0.084145	0.567035	16.89085	3.96E-05	-78.490905	0.044785
173	0.632019	2.056024	0.307399	1.149574	6.546658	0.010508	-178.93144	0.373832
174	0.206639	0.628511	0.328775	0.437313	4.233384	0.039636	-88.923887	0.04407
175	0	2.692625	0	0.659504	22.5497	2.05E-06	-47.05501	0
176	0	1.966993	0	0.532066	29.14751	6.71E-08	-54.873978	0
177	0.233764	0.735176	0.31797	0.487183	4.382567	0.036308	-109.41234	0.125968
178	0.369289	1.573325	0.234719	0.883237	8.741808	0.00311	-112.87973	0.05912
179	0	1.364013	0	0.587077	18.79019	1.46E-05	-45.497349	0
180	0.085342	1.422992	0.059974	0.950779	16.96929	3.80E-05	-100.29879	0
181	0.156983	1.566745	0.100197	0.928814	15.48947	8.30E-05	-115.99597	0
182	0.363321	1.907745	0.190445	0.946121	11.3975	0.000735	-127.67232	0.11977
183	0	1.786195	0	0.742005	33.66589	6.54E-09	-81.333522	0
184	0	0.408105	0	0.234142	11.69014	0.000628	-58.631005	0.029639
185	0	1.317178	0	0.510009	17.02862	3.68E-05	-57.485829	0.024626
186	0	1.075181	0	0.583226	24.49535	7.45E-07	-74.132034	0
187	0	0.860082	0	0.652051	14.43629	0.000145	-99.094543	0
188	0	0.875537	0	0.465653	23.14313	1.50E-06	-70.51044	0
189	0	0.626825	0	0.294302	15.72966	7.31E-05	-56.566508	0.014638
190	0	2.650027	0	0.532865	31.50378	1.99E-08	-50.806217	0
191	0	3.934745	0	0.834362	30.59364	3.18E-08	-51.832244	0
192	0	0.761543	0	0.403778	15.48476	8.32E-05	-58.23566	0.019555
193	0	2.80154	0	0.658196	40.28989	2.19E-10	-58.40126	0
194	0	1.440061	0	0.666099	30.71442	2.99E-08	-77.679159	0
195	0	1.898695	0	0.735113	39.7072	2.95E-10	-87.285932	0
196	0	0.664946	0	0.528478	10.88893	0.000967	-87.080431	0
197	0	0.549901	0	0.275258	14.42107	0.000146	-46.475782	0
198	0	1.738965	0	0.678587	32.34696	1.29E-08	-62.687484	0
199	0	0.967886	0	0.410247	22.56669	2.03E-06	-63.235707	0
200	0	0.888534	0	0.38005	20.88681	4.87E-06	-45.543771	0
201	0	0.749597	0	0.423003	17.31871	3.16E-05	-66.168268	0
202	0	1.002135	0	0.483133	24.97858	5.80E-07	-65.761892	0
203	0	1.84344	0	0.767879	34.86151	3.54E-09	-90.19202	0
204	0	0.99609	0	0.433964	20.7758	5.16E-06	-53.945804	0.016594
205	0	1.831105	0	0.575578	32.4467	1.23E-08	-55.996012	0
206	0	2.006087	0	1.265163	26.23018	3.03E-07	-122.68565	0.028698
207	0	1.440845	0	0.627299	33.27344	8.01E-09	-73.274007	0

208	0	2.930497	0	0.722062	40.25862	2.22E-10	-54.287288	0
209	0	0.947937	0	0.51648	23.34108	1.36E-06	-81.102948	0
210	0	1.481835	0	0.663896	30.7838	2.88E-08	-55.785658	0
211	0	0.939803	0	0.493768	22.44712	2.16E-06	-63.853681	0
212	0	3.559893	0	0.737863	38.92588	4.40E-10	-58.542259	0
213	0	0.87554	0	0.648453	14.42314	0.000146	-94.1445	0
214	0	1.100485	0	0.530674	23.52074	1.24E-06	-86.823506	0.033793
215	0	2.19335	0	0.873237	41.14557	1.41E-10	-96.231535	0.016829
216	0	1.243673	0	0.595649	29.25585	6.34E-08	-84.019863	0
217	0	0.648623	0	0.324309	15.00293	0.000107	-46.853379	0
218	0	1.372274	0	0.547936	30.11625	4.07E-08	-48.776387	0
219	0	0.619001	0	0.32041	14.37341	0.00015	-49.106822	0
220	0	1.31352	0	0.512911	25.21585	5.13E-07	-45.972844	0
221	0	0.429596	0	0.246376	9.300358	0.002291	-39.370478	0
222	0	0.61638	0	0.308993	16.09301	6.03E-05	-49.024512	0
223	0	1.413566	0	0.679555	31.91265	1.61E-08	-100.659	0
224	0	1.366916	0	0.786052	21.78366	3.05E-06	-81.337124	0
225	0	1.235677	0	0.566011	25.38168	4.70E-07	-75.141795	0.017147
226	0	2.245077	0	0.744331	37.06827	1.14E-09	-53.900921	0
227	0	0.135013	0	0.08081	4.046648	0.044259	-31.144134	0
228	0	1.172607	0	0.761058	19.50479	1.00E-05	-111.53456	0
229	0	0.928863	0	0.445325	20.59435	5.68E-06	-62.252843	0.050703
230	0	0.694781	0	0.325089	15.4625	8.42E-05	-41.603034	0
231	0	0.85757	0	0.643214	14.43006	0.000145	-101.56041	0
232	0	0.567017	0	0.316329	15.80661	7.02E-05	-52.055657	0
233	0	3.38305	0	0.56955	36.60017	1.45E-09	-46.78241	0
234	0	1.054609	0	0.530748	20.48595	6.01E-06	-50.676051	0
235	0	0.919473	0	0.508422	24.73817	6.57E-07	-67.304611	0
236	0	1.406321	0	0.48638	28.24222	1.07E-07	-51.753698	0
237	0	0.883992	0	0.392227	21.62748	3.31E-06	-42.867855	0
238	0	0.507832	0	0.376252	8.911973	0.002833	-68.723539	0
239	0	1.344534	0	0.825077	19.70565	9.03E-06	-103.49541	0
240	0	1.138509	0	0.45498	24.78533	6.41E-07	-47.903254	0
241	0	1.146962	0	0.489712	26.47478	2.67E-07	-57.562065	0
242	0	0.345489	0	0.191753	10.13011	0.001459	-49.524111	0.01419
243	0.099161	1.10174	0.090004	0.799873	10.90046	0.000961	-107.37593	0
244	0	0.878032	0	0.443519	19.52231	9.94E-06	-64.518958	0
245	0.157409	1.149442	0.136944	0.650302	14.05499	0.000178	-91.116786	0
246	0.050086	0.838525	0.059731	0.390548	14.07035	0.000176	-74.64441	0.06474
247	0	0.936491	0	0.56119	19.25173	1.15E-05	-79.221575	0
248	0	1.361217	0	0.547719	26.57119	2.54E-07	-51.179711	0
249	0	1.35228	0	0.800198	26.37836	2.81E-07	-102.98915	0.022222
250	0	0.966934	0	0.526837	25.06247	5.55E-07	-69.575635	0
251	0	0.357136	0	0.187075	9.768711	0.001775	-41.295229	0
252	0.058186	1.030715	0.056452	0.517196	15.95364	6.49E-05	-108.22338	0.08667
253	0.134047	1.665846	0.080468	0.688679	15.77766	7.12E-05	-84.860759	0.054999
254	0.467408	1.279374	0.365341	0.779593	4.291052	0.038313	-178.00275	0.393112
255	0	0.842525	0	0.400348	18.01392	2.19E-05	-68.244256	0
256	0.058533	0.781322	0.074915	0.44894	12.55987	0.000394	-150.29338	0.456795
257	0.272283	1.33654	0.203722	0.752304	10.77591	0.001028	-159.77808	0.278817
258	0	1.106312	0	0.528423	23.48662	1.26E-06	-62.028302	0
259	0	1.650213	0	0.387407	21.89747	2.88E-06	-40.647519	0
260	0	1.281652	0	0.851286	19.59714	9.56E-06	-106.34882	0
261	0	0.941426	0	0.415963	23.08961	1.55E-06	-60.588117	0

262	0	0.439184	0	0.213757	11.79832	0.000593	-61.347199	0.04429
263	0.334096	1.068007	0.312822	0.656138	5.57599	0.018208	-159.88277	0.321353
264	0.157101	2.245036	0.069977	0.729209	21.35428	3.82E-06	-73.493994	0
265	0	1.014677	0	0.732551	15.39556	8.72E-05	-99.775544	0
266	0	2.406939	0	0.564019	35.15798	3.04E-09	-52.984507	0
267	0.049233	0.852313	0.057764	0.480328	17.01854	3.70E-05	-108.25288	0.159438
268	0	0.941547	0	0.481565	17.83516	2.41E-05	-45.272992	0
269	0	0.475993	0	0.264622	13.32841	0.000261	-54.558241	0.014629
270	0	1.150819	0	0.488488	26.38048	2.80E-07	-77.684921	0.014639
271	0	0.89691	0	0.590456	17.14096	3.47E-05	-81.507804	0
272	0	1.52018	0	0.818324	27.10946	1.92E-07	-105.83824	0
273	0	1.64644	0	0.75247	33.0305	9.07E-09	-81.405912	0
274	0.059855	2.168956	0.027596	0.864002	27.74459	1.38E-07	-125.62917	0.138677
275	0.431853	2.868542	0.150548	1.310123	15.33675	8.99E-05	-164.15507	0.212933
276	0	0.733174	0	0.34164	16.20398	5.69E-05	-41.585103	0
277	0	1.364065	0	0.762091	24.79348	6.38E-07	-93.174492	0
278	0	1.112953	0	0.586689	17.54177	2.81E-05	-50.659292	0
279	0.181216	1.093516	0.165718	0.67104	10.82963	0.000999	-122.78324	0.146408
280	0	0.7117	0	0.400237	15.52483	8.14E-05	-59.285914	0
281	0	0.236432	0	0.132199	6.773181	0.009254	-35.292945	0
282	0	1.298664	0	0.570936	27.32229	1.72E-07	-77.872209	0
283	0	4.442423	0	0.786202	36.92271	1.23E-09	-62.772042	0
284	0	2.396515	0	0.962043	42.81988	6.00E-11	-88.644781	0
285	0	0.913325	0	0.507471	19.47603	1.02E-05	-66.376011	0
286	0	1.262213	0	0.497211	24.42798	7.71E-07	-47.875932	0
287	0	1.187524	0	0.664254	24.97418	5.81E-07	-78.342795	0
288	0	0.897774	0	0.482885	18.2598	1.93E-05	-48.02525	0
289	0	1.689454	0	0.559304	30.98922	2.59E-08	-53.861442	0
290	0	2.974212	0	0.838009	27.47686	1.59E-07	-54.387969	0
291	0	1.586564	0	1.035016	21.67879	3.22E-06	-127.01319	0.056902
292	0	1.556899	0	0.638231	32.60401	1.13E-08	-56.906376	0
293	0	1.417699	0	0.569869	25.74508	3.90E-07	-51.688454	0
294	0	1.015531	0	0.752796	16.54787	4.74E-05	-107.21857	0
295	0	1.274914	0	0.668249	28.08401	1.16E-07	-88.808118	0.016717
296	0	0.621315	0	0.363125	10.68245	0.001082	-42.195961	0
297	0	1.866734	0	0.394815	17.83504	2.41E-05	-38.356706	0
298	0	0.711361	0	0.322658	17.53903	2.81E-05	-50.47611	0.014716
299	0	1.312095	0	0.558414	26.44106	2.72E-07	-50.323453	0
300	0	1.741089	0	1.053272	29.07349	6.97E-08	-113.08321	0
301	0	1.359786	0	0.659428	28.64965	8.67E-08	-74.722525	0
302	0	1.410344	0	0.933515	19.55862	9.76E-06	-129.33617	0.090842
303	0	0.638517	0	0.336175	17.79405	2.46E-05	-60.511501	0
304	0	0.898026	0	0.693194	14.38198	0.000149	-111.54927	0
305	0	0.647674	0	0.343835	17.44402	2.96E-05	-62.719306	0
306	0.217799	0.719922	0.302531	0.625167	3.387717	0.065684	-120.81182	0.060183
307	0	0.867669	0	0.448487	19.6328	9.38E-06	-69.710932	0.016699
308	0.054367	1.025054	0.053038	0.553128	17.8191	2.43E-05	-87.298654	0
309	0	0.408169	0	0.195766	6.299484	0.012077	-25.859557	0
310	0	1.310541	0	0.524155	23.80753	1.06E-06	-50.689707	0
311	0.204232	0.773827	0.263925	0.500254	6.490107	0.010848	-104.96397	0.110836
312	0	0.329597	0	0.191333	9.568523	0.001979	-37.308315	0
313	0	0.838237	0	0.537526	18.0264	2.18E-05	-79.485814	0
314	0	1.358283	0	0.617585	27.19799	1.84E-07	-79.227875	0
315	0	1.092029	0	0.463037	25.61535	4.17E-07	-54.910683	0.014439

316	0	0.576566	0	0.273535	14.63882	0.00013	-47.261201	0
317	0	0.608483	0	0.352029	12.63897	0.000378	-67.177874	0.054837
318	0	0.519791	0	0.295999	14.67452	0.000128	-55.270399	0.014675
319	0	1.105746	0	0.708835	19.6692	9.21E-06	-90.803002	0
320	0	0.948098	0	0.582457	19.77285	8.72E-06	-78.576381	0.02185
321	0	0.703793	0	0.386002	16.58233	4.66E-05	-66.512313	0
322	0	1.241201	0	0.517085	24.07369	9.27E-07	-79.248642	0
323	0	0.668141	0	0.393472	10.74647	0.001045	-38.975015	0
324	0	0.942152	0	0.575195	17.74236	2.53E-05	-108.41775	0.174658

Note: The positively selected residue is coloured red.

Table S3

Primer	Location	Sequence (5′ to 3′)
F1	169	GAGGAGCAGGTATCAGGCA
R1	1471	CACCAAGTTCGGTAGGTTTAT
F2	1314	ATGATTTAGCCAGCACCA
R2	2596	GTTGAACAAACGAACCCTTA
F3	2373	AAGTGGACTGGGATAAACCCTA
R3	3299	TTGTCTGGGCTACTGCTCG
F4	3066	GCCCCTCTACATCATCCCC
R4	4119	GCAAAGGTTAAGGTGGTTCC
F5	3954	ACTCCTGGTGCTTCCTCTACA
R5	5137	GCTTTGAAGGCTCTTGGTCT
F6	4949	GCTATAACCCTTACCATCTCC
R6	6177	TGCTGGTAAAGAATAGGGTC
F7	5900	GCATCCGTTGACCTAACTATCTT
R7	7101	AAGGAGGTGACAGAGTGGCTATG
F8	6900	AAGCCTTCGCTGCTAAAC
R8	8036	TGGTTGGGATGATGGTAAGAA
F9	7924	CAAAGAATGGTGCCTCCC
R9	9118	GCCAGGCTTGAATGGTAAA
F10	8745	TCAAGCCTATGTATTTGTCCT
R10	10054	ACCATAGTTATCTGAGCCGA
F11	9799	CGACTCCCATTCTCCTTACG
R11	11164	TAAGGCTAAAATAATAAAGGGG
F12	11008	TTGACTACCCAAAGCCCA
R12	12256	CTTAGAGGGCAATAGGTGTAA
F13	12166	TGACACTGAATAAATACAGCCCT
R13	13187	TTTAGGTAAGAGGTATTTAGTGC
F14	13003	GGTTCCATTATTCACAGCCT
R14	14272	TAGGGTTAGTTGCTGTGGC
F15	14080	AATGGCTCAGCAGCTAAAG
R15	15431	TAATAAATGGGTGTTCTACTGG
F16	15207	TCCTCTTTGCCTATGCCAT
R16	16357	GATTTGCTGAGCGTAGGG
F17	15997	ATTATTCCTTGCATCTGGCT
R17	425	ACACTCAGGAGCGTATGA

Table S4

Family	Species name	Accession number	Mitogenome size (bp)
Outgroups			
Catostomidae	*Carpiodes carpio*	AY366087	16,611
	Catostomus commersonii	AB127394	16,625
	Cycleptus elongatus	AB126082	16,626
	Erimyzon oblongus	AP011228	16,627
	Hypentelium nigricans	AB242169	16,625
	Ictiobus bubalus	AP009316	16,612
	Ictiobus cyprinellus	KP306894	16,611
	Minytrema melanops	DQ536432	16,627
	Moxostoma congestum	AP009317	16,626
	Moxostoma poecilurum	AB242167	16,637
	Myxocyprinus asiaticus	AP006764	16,636
	Xyrauchen texanus	EU265776	16,634
Gyrinochelidae	*Gyrinocheilus aymonieri*	AB242164	16,609
Ingroups			
Botiidae	*Chromobotia macracanthus*	AB242163	16,937
	Leptobotia elongata	JQ230103	16,591
	Leptobotia mantschurica	AB242170	16,588
	Leptobotia microphthalma	KC865424	16,512
	Leptobotia rubrilabris	KF534784	16,585
	Leptobotia taeniops	KM386686	16,592
	Parabotia banarescui	KM393222	16,590
	Parabotia fasciata	KM393223	16,590
	Sinibotia superciliaris	JX155735	16,572
Vaillantellidae	*Vaillantella maassi*	AB242173	17,296
Cobitidae	*Acantopsis choirorhynchos*	AB242161	16,600
	Cobitis choii	EU656112	16,566
	Cobitis elongatoides	KF926686	16,541
	Cobitis lutheri	AB860297	16,639
	Cobitis melanoleuca granoei	KF908768	16,636
	Cobitis sinensis	AY526868	16,553
	Cobitis striata	AB054125	16,572
	Cobitis takatsuensis	AP009306	16,647
	Koreocobitis naktongensis	HM535625	16,567
	Koreocobitis rotundicaudata	JN607253	16,571
	Misgurnus anguillicaudatus	DQ026434	16,565
	Misgurnus bipartitus	KF562047	16,636
	Misgurnus mohoity	KF386025	16,566
	Misgurnus nikolskyi	AB242171	16,570
	Niwaella delicata	AP009308	16,571
	Pangio anguillaris	AB242168	16,602
	Paramisgurnus dabryanus	KJ027397	16,570

Balitoridae			
	Formosania lacustris	M91245	16,558
	Jinshaia abbreviata	KJ754936	16,567
	Jinshaia sinensis	KJ739867	16,567
	Lepturichthys fimbriata	KJ830772	16,567
	Metahomaloptera omeiensis	KM077128	16,558
	Plesiomyzon baotingensis	KF732713	16,541
	Sewellia lineolata	AP011292	16,522
	Sinogastromyzon puliensis	FJ605359	16,551
	Sinogastromyzon sichangensis	KF711948	16,567
	Vanmanenia pingchowensis	KP005457	16,560
	Homaloptera leonardi	AB242165	16,580
Nemacheilidae	*Barbatula nuda*	KF574248	16,619
	Barbatula toni	AB242162	16,617
	Barbatula labiata	**KT192057**	**16,584**
	Barbatula sp.	**KT259193**	**16,578**
	Homatula potanini	KM017732	16,569
	Homatula variegata	JX144893	16,571
	Lefua echigonia	AB054126	16,559
	Schistura balteata	AB242172	16,564
	Schistura longus	**KT213583**	**16,582**
	Triplophysa anterodorsalis	KJ739868	16,567
	Triplophysa bleekeri	JX135578	16,568
	Triplophysa robusta	KM406486	16,570
	Triplophysa rosa	JF268621	16,585
	Triplophysa siluroides	KJ781206	16,574
	Triplophysa stoliczkai	JQ663847	16,571
	Triplophysa strauchii	KP297875	16,590
	Triplophysa aliensis	**KT213584**	**16,565**
	Triplophysa alticeps	**KT213585**	**16,572**
	Triplophysa sp.1	**KT213586**	**16,567**
	Triplophysa sp.2	**KT213587**	**16,574**
	Triplophysa brevicauda	**KT213588**	**16,572**
	Triplophysa chondrostoma	**KT213589**	**16,568**
	Triplophysa dalaica	**KT213590**	**16,576**
	Triplophysa dorsalis	**KT213591**	**16,572**
	Triplophysa hsutschouensis	**KT213592**	**16,571**
	Triplophysa leptosoma	**KT213593**	**16,570**
	Triplophysa markehenensis	**KT213594**	**16,569**
	Triplophysa microps	**KT213595**	**16,571**
	Triplophysa minxianensis	**KT213596**	**16,574**
	Triplophysa moquensis	**KT213597**	**16,571**
	Triplophysa nujiangensa	**KT213598**	**16,570**
	Triplophysa orientalis	**KT213599**	**16,570**
	Triplophysa pappenheimi	**KT213600**	**16,572**
	Triplophysa pseudostenrua	**KT213601**	**16,638**
	Triplophysa scleroptera	**KT213602**	**16,570**

Triplophysa siluroides	**KT213603**	**16,574**
Triplophysa stenura	**KT213604**	**16,568**
Triplophysa stewarti	**KT213605**	**16,567**
Triplophysa sp.3	**KT259195**	**16,562**
Triplophysa strauchii ulacholicus	**KT259194**	**16,568**
Triplophysa tenuis	**KT224363**	**16,571**
Triplophysa tibetana	**KT224364**	**16,574**
Triplophysa wuweiensis	**KT224365**	**16,681**
Triplophysa xichangensis	**KT224366**	**16,570**
Hedinichthys yarkandensis	**KT224367**	**16,562**

Note: Sequences in bold were newly determined in this study.

Table S5

Subset for 13 PCGs	Best model	Partition scheme
1	GTR+I+G	ND4_1st, ND4L_1st, ND5_1st, ND6_1st, ATP6_1st, ATP8_1st, ATP8_2nd, ATP8_3rd, COX1_1st, COX2_1st, COX3_1st, CYTB_1st, ND1_1st, ND2_1st, ND3_1st
2	GTR+I+G	ND4_2nd, ND4L_2nd, ND5_2nd, ND6_2nd, ATP6_2nd, COX1_2nd, COX2_2nd, COX3_2nd, CYTB_2nd, ND1_2nd, ND2_2nd, ND3_2nd
3	GTR+I+G	ND4_3rd, ND4L_3rd, ND5_3rd, ATP6_3rd, COX1_3rd, COX2_3rd, COX3_3rd, CYTB_3rd, ND1_3rd, ND2_3rd, ND3_3rd
4	GTR+G	ND6_3rd

Figure S1

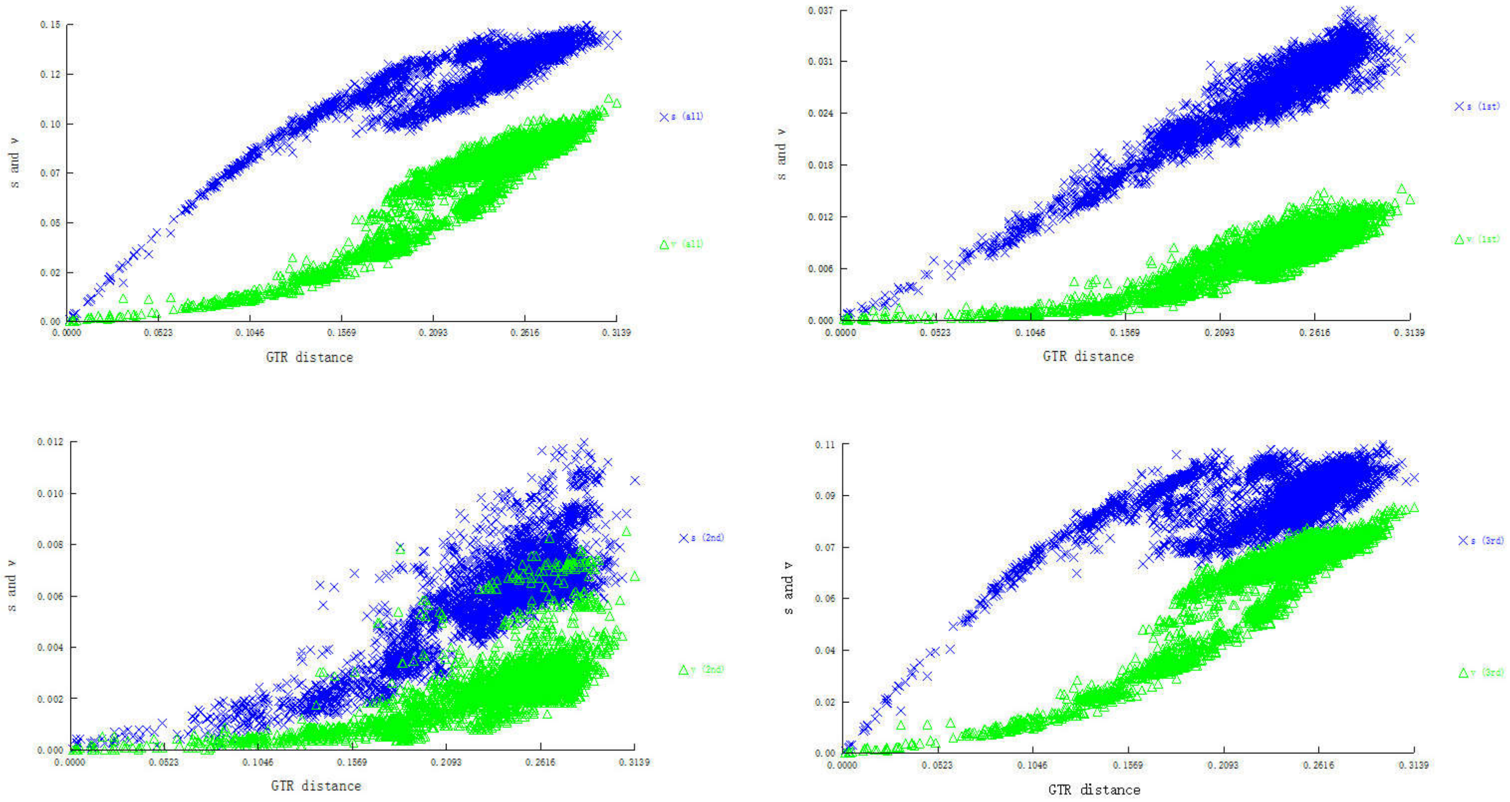

Supplement: Supplementary Information [file srep29690-s1.pdf]
